# Implementation of Relativistic Coupled Cluster Theory for Massively Parallel GPU-Accelerated Computing Architectures

**DOI:** 10.1021/acs.jctc.1c00260

**Published:** 2021-08-09

**Authors:** Johann V. Pototschnig, Anastasios Papadopoulos, Dmitry I. Lyakh, Michal Repisky, Loïc Halbert, André Severo Pereira Gomes, Hans Jørgen Aa Jensen, Lucas Visscher

**Affiliations:** † Department of Chemistry and Pharmaceutical Sciences, Faculty of Science, Vrije Universiteit Amsterdam, de Boelelaan 1083, 1081 HV Amsterdam, The Netherlands; ‡ National Center for Computational Sciences, Oak Ridge National Laboratory, Oak Ridge, Tennessee 37831, United States; § Hylleraas Centre for Quantum Molecular Sciences, Department of Chemistry, UiT The Arctic University of Norway, N-9037 Tromsø, Norway; ∥ Universite de Lille, CNRS, UMR 8523 − PhLAM − Physique des Lasers, Atomes et Molecules, F-59000 Lille, France; ⊥ Department of Physics, Chemistry and Pharmacy, University of Southern Denmark, DK-5230 Odense M, Denmark

## Abstract

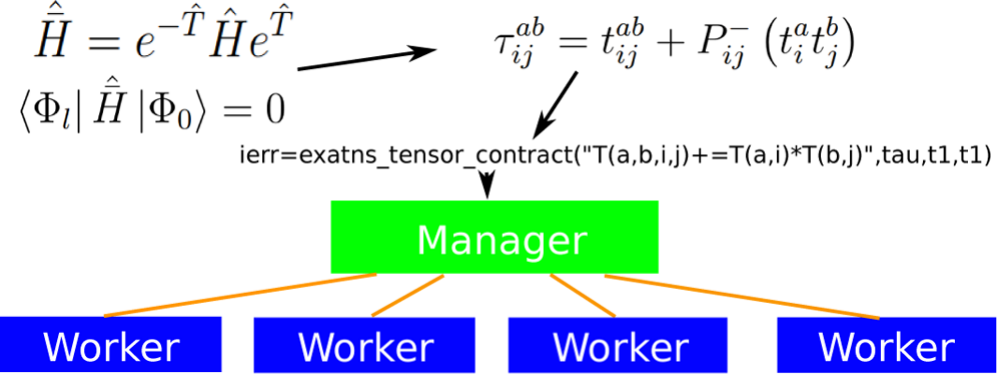

In this paper, we report reimplementation of the core algorithms of relativistic coupled cluster theory aimed at modern heterogeneous high-performance computational infrastructures. The code is designed for parallel execution on many compute nodes with optional GPU coprocessing, accomplished via the new ExaTENSOR back end. The resulting ExaCorr module is primarily intended for calculations of molecules with one or more heavy elements, as relativistic effects on the electronic structure are included from the outset. In the current work, we thereby focus on exact two-component methods and demonstrate the accuracy and performance of the software. The module can be used as a stand-alone program requiring a set of molecular orbital coefficients as the starting point, but it is also interfaced to the DIRAC program that can be used to generate these. We therefore also briefly discuss an improvement of the parallel computing aspects of the relativistic self-consistent field algorithm of the DIRAC program.

## 1 Introduction

Computational chemistry is a standard tool in the analysis, design, and synthesis of molecular systems.^1^ In particular, density functional theory (DFT) is used in a routine fashion in academic and industrial applications. While often sufficiently accurate, DFT does not allow for molecule-specific validations of the accuracy of its predictions. However, this is possible for the wave-function-based methods, such as coupled cluster (CC) theory, for which extensions of the single-particle basis combined with an increase of the excitation level in the CC ansatz lead to a systematic improvement of the accuracy. For organic molecules, CC methods can nowadays predict molecular structures to a precision that is better than 1 pm in bond lengths and better than one degree in bond angles.^2,3^ Furthermore, the efficient equation-of-motion (EOM) treatment of electronically excited states^4,5^ makes it possible to study photochemical processes and aid the interpretation of spectroscopic data. The standard approaches to compute ground-state energies, molecular properties, and electronically excited states have all been generalized to relativistic theory as well, yielding methods that can provide very high accuracy in the electronic structure part of a calculation. This is demonstrated in numerous small-molecule applications^6−10^ for which steep scaling with the system size of the coupled cluster algorithm is not an issue. This rapid increase in computational requirements does, however, in practice, prevent the application of relativistic CC with a fully spin–orbit-coupled reference wave function to systems that contain more than about ten atoms. In nonrelativistic CC computations, it is, of course, possible to go to larger system sizes, but relativistic corrections can then only be incorporated approximately (usually to some order in perturbation theory and often in combination with effective core potentials). With the current implementation, we want to enable the treatment of larger molecular systems with an all-electron correlated relativistic method that can be used to estimate the accuracy of different approximations for systems with significant correlation and relativistic effects.

Further improvements of the relativistic algorithms are well possible, however, as many reduced-scaling techniques from nonrelativistic algorithms can be taken over in a slightly modified form. One example is the use of density fitting (DF)^11−13^ or Cholesky decomposition^14−17^ to reduce the size of the two-electron integral tensors. Here, relativistic treatments require handling of the density of small components of the Dirac wave functions or, equivalently, fitting of the relativistic correction terms to a two-electron operator in the two-component formulation.^18^ Another example is the use of the Laplace transform in Møller–Plesset perturbation theory,^17^ where the effects of spin–orbit coupling are visible in the form of (quaternion) imaginary contributions to the density matrices. In both examples, one observes a steep increase in the computational cost of the algorithm but also notes that formal scaling with the system size is identical to that of the nonrelativistic algorithm. Because numerically small contributions to tensor elements can be neglected by the use of screening techniques and many additional terms are only significant in the vicinity of heavy atoms, scaling can in principle be further improved. On the other hand, one may observe that inclusion of only a single heavy atom already presents a challenge due to the number of electrons that has to be correlated in a coupled cluster treatment. This can be illustrated by comparing the CO_2_ molecule to the uranyl ion, UO_2_^2+^. Both are linear triatomic systems with the oxygen atoms contributing a total of 12 valence electrons. In CO_2_, this yields a total of 16 valence electrons that are to be correlated, while in uranyl, one needs to correlate at least 24 electrons^19^ and preferably 34 electrons^20^ due to the large uranium atom.

Both the increase of the number of electrons to be correlated and the switch from real to complex algebra make relativistic calculations rather demanding. However, they are very well suited for deployment on supercomputers because the key algorithms can be formulated as contractions of large tensors, which can be carried out with a relatively high computational efficiency. To be able to realize the full potential of both reduced-scaling techniques and parallel computing, as is nowadays common^21^ in non- or scalar-relativistic approaches, it is advantageous to first create a modern implementation of the relativistic coupled cluster algorithm.

The legacy CC code of DIRAC, RELCCSD,^22^ allows for parallelization^23^ but does not scale well on a larger number of nodes as it was designed for clusters of the early 2000s. An advantage of this code is the use of spatial symmetry, which reduces the computational cost and is helpful in interpreting molecular spinors and electronic transitions. Both aspects are less relevant when applying the coupled cluster approach to large molecular systems that possess (almost) no symmetry. In our reimplementation, we therefore do not consider molecular symmetries but instead focus on data and compute parallelism. The ExaCorr implementation that we describe here is based on the ExaTENSOR library,^24^ a distributed numerical tensor algebra library for GPU-accelerated HPC platforms developed at the Oak Ridge Leadership Computing Facility (OLCF).

The main body of post-Hartree–Fock quantum–chemical machinery is based on numerical tensor algebra. For the commonly used coupled cluster singles and doubles (CCSD) model, it is possible to formulate^25^ all operations as tensor contractions of at most four-dimensional tensors. This strict adherence to formulation in terms of tensor contractions is the key to computationally efficient implementation that we present here. It should be regarded both as a platform for future developments and a tool to generate reference data to validate approximate methods in which a large number of two-electron integrals and/or excitation amplitudes are reduced by, for instance, rank reduction^26,27^ or Laplace transformation.^28^

The paper is structured in the following way. In Section 2, we briefly summarize the coupled cluster algorithms that we consider in the current work. This is followed by Section 3 in which the implementation of the algorithms is discussed. Section 4 is devoted to the details of the computations we used to test the implementation. In Section 5, we present calculations for validation of the correctness of the results by comparing with the reference RELCCSD implementation as well as calculations aimed at showing the computational scaling. The conclusion follows, which includes a discussion of the follow-up work.

## 2 Theory

### 2.1 Relativistic Theory

A prerequisite for a relativistic coupled cluster calculation is a set of two- or four-component molecular spinors obtained by solving the relativistic Dirac–Hartree–Fock equation. In the four-component case, this equation reads
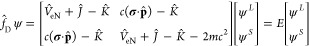
1in which **σ** is the vector of the 2 × 2 Pauli matrices and ψ*^S^* and ψ*^L^* are the small and large component parts of the full 4-spinor ψ, respectively. Each of these is itself a 2-spinor with as individual components ψ_α_^*X*^ and ψ_β_^*X*^ (*X* = *L*, *S*). *V̂*_eN_ represents the nuclear–electron interaction, usually defined with a Gaussian model of the nuclear charge distribution,^29^ and the local *Ĵ* and nonlocal *K̂* operators describe the electron–electron interaction in the mean-field approximation
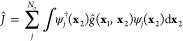
2
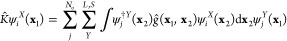
3where *N*_o_ denotes the number of occupied spinors. The two-electron interaction operator *ĝ*(1, 2) is the Coulomb(−Gaunt) operator
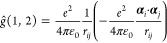
4Before proceeding to the coupled cluster stage, all operators are transformed using the exact two-component (X2C) method that allows re-expression of the four-component spinors into a two-component picture. Three main variants of the X2C method are used in the current work. The first one, termed X2C-1e, is based on the simple X2C transformation of the one-electron Dirac Hamiltonian that is combined with the nonrelativistic Coulomb operator to describe the electron–electron interactions.^30,31^ Since X2C-1e omits all two-electron relativistic corrections and leaves the relativistic scalar and spin–orbit coupling operators associated with the nuclear potential unscreened, the second variant extends X2C-1e by an explicit addition of the atomic mean-field two-electron potential (done via the AMFI code^32^). This approach is termed X2C-AMFI and is the default X2C Hamiltonian in DIRAC. In both X2C approaches, the transformation to the two-component picture is carried out before the Hartree–Fock procedure, and therefore, the two-electron molecular integrals that involve small component basis functions are never computed. In contrast, these types of integrals do enter in the third variant, named X2Cmmf,^18^ as the X2C transformation is carried out after solving the Hartree–Fock equations, and therefore, the full molecular potential is used to define the X2C transformation. This makes X2Cmmf more accurate than the X2C-AMFI (or X2C-1e) approach. Moreover, the two-electron spin–orbit contributions of electrons that will not be explicitly taken into account in the correlation treatment (hereafter referred to as the core or frozen electrons) are treated exactly.

Although (obviously) more expensive due to the mean-field part of the calculations, the X2Cmmf procedure has the same favorable computational characteristics as the X2C-AMFI (or X2C-1e) procedure in the post-HF steps, with the advantage that it yields results that are very close to the full four-component treatment.^5,18^ In the current implementation, the X2Cmmf approach functions as the high-level reference method, while in the DIRAC code, the use of X2C-AMFI and a nonrelativistic treatment (to compare with other coupled cluster implementations) is also supported. Currently, the X2Cmmf approach also allows for an approximate inclusion of the Gaunt interaction,^18^ and an implementation of the full Dirac–Coulomb–Gaunt operator for use in very precise benchmark calculations is planned as well.

All of the aforementioned methods apply the no-virtual pair approximation such that the Hamiltonian to be used for the coupled cluster treatment is written in the second quantization as
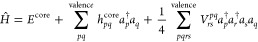
5with *V*_*rs*_^*pq*^ being the antisymmetrized two-electron integrals
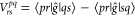
6and valence in the summation indicating the spinors that are active in the coupled cluster calculation (omitting frozen occupied spinors as well as deleted virtual spinors). The *E*^core^ constant contains the energy of the core electrons as well as the nuclear repulsion term. The operator *h*^core^ describes the interaction between the frozen core electrons and the valence electrons and contains the Dirac kinetic energy and nuclear–electron interaction terms defined above as well. The main difference with the nonrelativistic treatments that use an identical expression, is the fact that the tensors **h**^core^ and **V** are defined in complex algebra, whereas in the nonrelativistic treatments, it is usually possible to employ real algebra.

In nonrelativistic quantum chemistry, the Hamiltonian is spin-free, which makes it possible to separate the spatial and spin degrees of freedom and solve equations for spatial orbitals. In relativistic computations, such a separation is not possible because relativistic spinors cannot be written as a simple product of a spatial and spin function. However, in the absence of magnetic fields, one may still use time-reversal symmetry, also known as Kramers symmetry, as each spinor can be related to another with the same energy.^33,34^ Use of this symmetry implies a Kramers-restricted (KR) algorithm in which the occupation of each of the two spinors that comprise a Kramers pair is kept identical in defining the mean-field potential. In contrast, a Kramers-unrestricted (KU) algorithm treats the spinors independent of each other and allows obtaining the so-called spin polarization effect.^35^

As the use of Kramers symmetry has little advantage in coupled cluster calculations,^22,36^ and we intend to keep the implementation modular and independent of the program used to generate the spinors, we will henceforth assume that all spinors are unrelated to each other, demanding only orthogonality between them. The consequence of these choices is an increase of the amount of data that needs to be dealt with: in a nonrelativistic restricted CCSD algorithm, the largest tensor appearing in the amplitude equations is of size *n*_vir_^4^ (with *n*_vir_ being the number of the virtual spatial orbitals), while in the relativistic treatment, this increases to 16 or 256 *n*_vir_^4^ complex numbers, with a corresponding increase in memory requirements and the number of floating point operations to generate and contract this tensor.

### 2.2 Coupled Cluster Algorithms

The wave function in the coupled cluster method is defined as
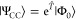
7where |Φ_0_⟩ is the single-determinant wave function. The cluster operator *T̂* is most commonly restricted to the single and double hole–particle excitations
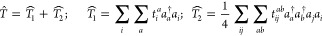
8defining the coupled cluster singles and doubles method (CCSD). The energy and cluster amplitudes are computed using equations
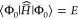
9
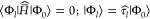
10with  denoting a generic excitation operation yielding any (singly, doubly, etc.) excited determinant |Φ_*l*_⟩, and where the similarity-transformed Hamiltonian
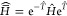
11is employed. The working equations for this formalism are well known and can for instance be found in the paper^22^ describing the RELCCSD program that we used as a reference implementation. In contrast to this code, for the current implementation, we assume that the working memory of the parallel computer is large enough to keep all tensors in memory. Furthermore, we formulate all operations as tensor contractions to enable efficient use of the ExaTENSOR library. Some intermediates were therefore also altered, resulting in the working equations listed in the Supporting Information. To allow for faster calculations, the CC2 approximation is implemented according to the working equations in the Supporting Information. To speed up the convergence of the CC solver, the direct inversion in the iterative subspace (DIIS) algorithm^37^ was implemented. Triple excitations are necessary to achieve chemical accuracy, but they are computationally expensive. A widely applied compromise is thus to add them perturbatively.^22,38,39^ The relevant working equations can be found in the Supporting Information.

To obtain first-order molecular electronic properties at the coupled cluster level, we use the Lagrange formalism,^40^ which requires solving the equations for the Lagrange multipliers {**λ**}
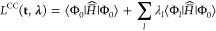
12

These are obtained from the stationary conditions
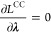
13
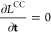
14where eq 13 represents the CC equations. Note that this definition of the Lagrangian neglects orbital relaxation, which is assumed to be partly covered by the  operator.^41^Equation 14 is solved to obtain the values of the Lagrange multipliers after which the expectation value of any one-body operator *Ô* can be computed by computing the one-body density matrix **γ**(41)
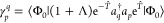
15
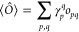
16The symmetrized one-body density matrix is transformed to the atomic orbital basis and then contracted with the matrix representation of the appropriate property operator *Ô*. The resulting working equations are listed in the Supporting Information.

## 3 Implementation

In this part, the details of the implementation are presented. To run coupled cluster computations, molecular spinors for a reference state are required. Section 3.1 describes the computation of these molecular spinors as well as highlights the changes required for improving the performance for larger molecular systems that have become feasible with the new implementation. The implementation utilizes two separate libraries to perform the compute-intensive operations. The calculation of two-electron integrals in the atomic basis is performed by the efficient InteRest library (Section 3.2), while the tensor contractions that comprise the majority of the coupled cluster algorithm are performed with the ExaTENSOR library (Section 3.3). Input handling and interfacing to the self-consistent field (SCF) programs are discussed in Section 3.4, while the transformation of two-electron integrals from the atomic to the molecular basis is described in Section 3.5. Finally, details regarding the coupled cluster code are discussed in Section 3.6.

### 3.1 Generation of the Molecular Spinors

Molecular spinors are required for the ExaCorr coupled cluster module, and they thus need to be efficiently generated for large system sizes. Because of the fast evaluation of two-electron integrals by the InteRest module, the efficient parallel implementation of the AO-to-MO transformation, and the fast solution of the CC equations described below, for DIRAC calculations, the Fock matrix diagonalizations required in the self-consistent field (SCF) stage became a bottleneck. As this step was not parallelized, it became excruciatingly slow for large AO basis spaces.

Historically, before DIRAC, the well-known double point groups as formulated by Wigner^42^ were used in the pioneering 4c relativistic molecular codes. When the SCF optimization was implemented in DIRAC,^34,43^ we used instead a more general quaternion description, which in fact relies on the simpler (single) point group irreps for quaternion basis function components.^43^ This implementation has, with small adjustments, been used until work on ExaCorr started, and each SCF-DIIS iteration has thus been based on a direct MPI parallel construction of Fock matrices based on the DALTON implementation,^44^ followed by a sequential quaternion generalization of the Fock matrix diagonalization (see Appendix D in ref (43)). This procedure gave satisfactory scaling with the number of MPI nodes for calculations of up to approximately 2000 AOs, which were feasible with the RELCCSD module. However, the new ExaCorr CC module described in this paper allows for larger applications and significantly large AO spaces and it became paramount that one should be able to do SCF calculations with 1000–5000 AO basis functions (and more in the future) in a small fraction of the wall time needed for the AO-to-MO transformation and the CC calculations. Analyzing the SCF performance for such larger systems with large numbers of compute nodes, it turned out that the parallel Fock matrix construction is acceptably efficient, but it was no surprise that the sequential quaternion matrix diagonalization needed in the MO-based DIIS algorithm required revision.

In this subsection, we describe how this diagonalization bottleneck was removed by tuning the sequential QDIAG code and addition of OpenMP structures in QDIAG. In relativistic quantum chemistry, there are mostly two approaches used for diagonalization. On the one hand, quaternions can be used to get matrix representation in real numbers, which is used here and in a recent publication dealing with large-scale quaternion matrix diagonalization.^45^ On the other hand, complex numbers and routines can be used, which are applied in ReSpect.

The implementation of the QDIAG routines in DIRAC by Saue^43^ in 1995 was based on his clever quaternion generalization of the complex diagonalization routines in EISPACK, where the EISPACK routines are direct transcriptions of the original ALGOL versions. However, ALGOL just as C and C++ uses row-major storage of matrices, while FORTRAN uses column-major storage. Therefore, the EISPACK routines were very inefficient for larger matrices because of many cache misses caused by the large strides in memory. A necessary first step was consequently to rewrite the QDIAG routines by transposing the access to all matrices followed by improvements of the logical structure. This change by itself already caused significant improvement in the sequential performance. The resulting implementation was then suitable for OpenMP parallelization. Initial timings of a large application on the TITAN supercomputer that was performed with 800 cores indicated that OpenMP parallelization with just eight OpenMP threads was sufficient to reduce the time spent in diagonalization to less than 11 min, compared to an overall wallclock time of 66 min in one SCF iteration (outputs of this and other benchmark runs are provided in a separate repository^46^). Additional timings on the SUMMIT supercomputer also showed that the wall time spent in diagonalization is much less than that needed for other steps like Fock matrix construction, and therefore, it was deemed unnecessary to also program additional GPU and/or MPI parallelization.

### 3.2 InteRest Integral Library

In the ExaTENSOR library (described below), it is possible to call an external library to initialize a particular tensor with the desired values. This mechanism allows for efficient parallel computation of the electron repulsion integrals (ERIs). A prerequisite is, however, that this external library is sufficiently modular, a requirement that could not be met by the legacy HERMIT integral generator used in DIRAC. We therefore interfaced the InteRest library^47^ to enable parallel computation of the ERIs arising from relativistic and nonrelativistic theories.

As discussed in ref (35), all commonly applied basis types in relativistic calculations are of multicomponent spinor nature and can uniformly be formulated in terms of real quaternion functions () or complex quaternion functions () over the field of real numbers . A product of any two quaternion basis functions *X*_*a*_(*r⃗*) and *X*_*b*_(*r⃗*) defines the so-called quaternion overlap distribution function Ω_*ab*_(*r⃗*) ≡ *X*_*a*_^†^(*r⃗*)*X*_*b*_(*r⃗*) in terms of which one can formulate and design an efficient algorithm for evaluation of nonrelativistic and relativistic ERIs.^35^ For instance, if refers to a restricted kinetically balanced (RKB) basis,^48^ then  comprises four real quaternion components. Then, a single quadruplet of ERIs defined similarly to the nonrelativistic case as
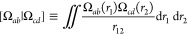
17requires the evaluation and processing of 25 times more real scalar integrals than in the nonrelativistic case. InteRest utilizes the Obara–Saika integration technique over Cartesian Gaussians^49^ to compute all of these scalar integrals in parallel and groups them into four integral classes [LL|LL], [SS|LL], [LL|SS], and [SS|SS] according to their values, which gradually decrease in powers of *c*^–2^.^35^ At the expense of going from real to complex quaternion functions, the presented uniform formalism for relativistic ERI evaluation can also be applied in the solid-state domain.^50^ Additional basis requirements needed for magnetic property calculations, such as the restricted magnetic balance^51^ (RMB) in combination with the gauge-including atomic orbitals (RMB-GIAO),^52^ can also be handled with the discussed integral scheme. A thorough discussion on this topic is given in ref (35).

### 3.3 ExaTENSOR and TAL-SH Backends

The ExaCorr module provides two distinct implementations of coupled cluster methods, one intended for execution on a single shared-memory node with an optional GPU acceleration and another one for execution on many such nodes (distributed parallelism), thus supporting a broad variety of computer platforms, from simple workstations to leadership HPC systems. Both implementations use the ExaTENSOR library^24^ as a massively parallel GPU-accelerated processing backend for numerical tensor algebra operations, although there are some differences in the interface between the single- and multinode API. For the single-node runs (with OpenMP multithreading and/or GPU acceleration), only the single-node component of ExaTENSOR, the TAL-SH library,^53^ is used. The single-node implementation is more efficient when MPI parallelization is not needed. It also serves as a validation reference for the corresponding multinode implementation. The ExaTENSOR library is written in a mix of Fortran-2008 and C/C++. It depends on BLAS, LAPACK, OpenMP, CUDA, and MPI (MPI is not necessary for single-node runs, while CUDA is only necessary for the GPU-enabled builds).

**Figure 1:**
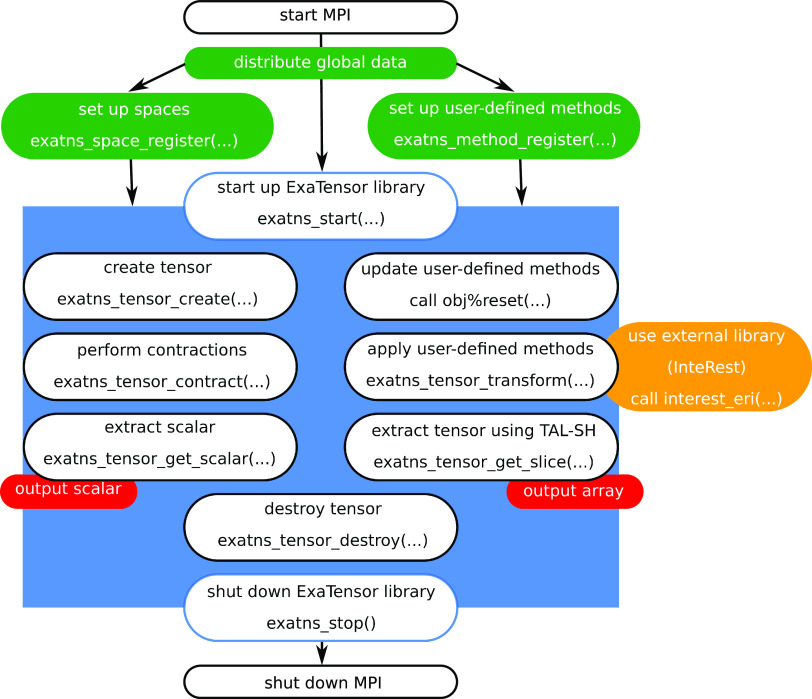
ExaCorr computational workflow based on the ExaTENSOR library.

Figure 1 outlines the computational workflow where the ExaCorr module offloads all computationally expensive operations (primarily tensor contractions) to the ExaTENSOR library. Essentially, the high-level interface of the ExaTENSOR library allows for the creation, destruction, addition, and contraction of distributed tensors via a single API call per operation, thus making it possible to directly translate tensor equations into the library calls. Such direct translation of quantum many-body equations into a human-readable code drastically accelerates the implementation of new coupled cluster methods in the ExaCorr module. Additionally, ExaTENSOR provides API for user-defined transformations on distributed tensors, which are often necessary in the coupled cluster algorithms. As described in Figure 1, the general computational workflow of a coupled cluster method implemented in ExaCorr starts with a replication of some global data, like molecular spinor coefficients, diagonal elements of the Fock matrix, etc., which normally do not consume much memory. Then, all vector spaces necessary for defining many-body tensors are explicitly registered, such as the space of atomic orbitals, occupied molecular spinors, virtual molecular spinors, etc. After that, all necessary ExaCorr-specific unary tensor transformations are registered as well. These extensions of the ExaTENSOR library are implemented as extensions of an abstract tensor transformation class provided by the ExaTENSOR interface. Once this is done, the ExaTENSOR parallel runtime (domain-specific virtual processor^24^) is started within a provided MPI communicator. After initialization, ExaTENSOR will begin accepting commands to perform distributed tensor algebra operations that realize a given coupled cluster algorithm. Importantly, the ability to implement user-defined tensor transformations facilitates the use of external libraries within ExaTENSOR, for example, the InteRest library,^47^ which was straightforwardly integrated with ExaTENSOR to enable parallel computation of the Coulomb integrals. Finally, once the given coupled cluster workload has been executed to completion, a local copy of the resulting scalar (e.g., energy, property) or tensor (e.g., density matrix) of interest can be retrieved. At the very end, the ExaTENSOR parallel runtime is explicitly shut down and control is handed back to the stand-alone ExaCorr or DIRAC program.

In ExaTENSOR, a tensor is formally defined as a multi-indexed vector from a linear space constructed as a direct product of basic (single-indexed) vector spaces. Such a multi-indexed vector (tensor) is represented by an array of complex numbers. The number of basic spaces in the direct product space defines the order of tensors living in that direct product space (note that in physics the tensor order is usually called the tensor rank). Each tensor dimension is thus associated with a specific basic space (or its subspace) from the defining direct product space. In practice, one must explicitly register all necessary basic vector spaces by calling the exatns_space_register API function provided by ExaTENSOR. To construct a basic vector space, one simply needs to provide a basis for that space or just specify its dimension. One can also construct a subspace of a registered basic vector space, thus enabling construction of tensor slices. Importantly, the definition of basic vector spaces requires their splitting into a number of subspaces chosen by the user, thus inducing the splitting of tensors into tensor slices. These slices are called elementary tensor blocks. All tensors are stored as distributed collections of such elementary tensor blocks. In the current implementation, the segment size used for splitting a basic vector space into a direct sum of its subspaces can be controlled by a keyword (see the Supporting Information).

Another prerequisite of coupled cluster algorithms is the necessity of custom tensor transformations (or initializations), like initialization of the Coulomb integral tensor, import of pre-existing many-body tensors (e.g., the Fock matrix), Jacobi preconditioning during amplitude updates, etc. Each such tensor initialization or transformation can easily be injected into ExaTENSOR by implementing user-defined tensor classes extending the abstract class tens_method_uni_t, followed by their registration with exatns_method_register. These user-defined subclasses can further be classified as either static or dynamic. The objects of static subclasses do not change their internal state after the registration with the ExaTENSOR runtime, whereas the objects of dynamic subclasses are allowed to change their internal state after the registration, thus enabling further flexibility and dynamic behavior during the execution of a tensor algorithm.

Once all necessary basic vector spaces/subspaces and user-defined tensor methods have been pre-registered, one may proceed to the execution of actual tensor operations on distributed tensors. A tensor is created via calling exatn_tensor_create, where a user provides which space/subspace each tensor dimension is associated with. Inside the ExaTENSOR parallel runtime, each tensor is recursively decomposed into smaller slices, which are distributed across all nodes (the tensor decomposition is induced by the direct sum structure of the vector spaces defining tensor dimensions). A tensor can then be initialized to either a scalar value or some custom value via a user-defined initialization method (exatns_tensor_init). There are three main tensor operations currently provided by ExaTENSOR: user-defined unary tensor transformation (exatns_tensor_transform), tensor addition (exatns_tensor_add), and tensor contraction (exatns_tensor_contract). These are sufficient for implementing the majority of coupled cluster algorithms. Both tensor addition and tensor contraction API take symbolic strings specifying the addition/contraction index pattern, for example
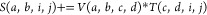
for a partial contraction over indices *c* and *d*, or
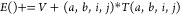
for a full contraction over all indices in which the complex conjugate values of the tensor *V* are used (indicated by the + symbol). Note that ExaTENSOR can handle arbitrary permutations of indices in tensor contraction specifications. Explicit tensor reordering is usually not necessary but can be achieved with the following permuted tensor addition specification
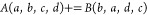
This reordering is employed in the creation of the antisymmetrized ERIs of eq 6 after the AO-to-MO transformation is completed.

In principle, tensor operations submitted to ExaTENSOR are processed asynchronously by all available MPI processes but one can also invoke bulk synchronization by calling exatns_sync to ensure the completion of all outstanding computations (a global barrier). Once all necessary computations have been completed, one can retrieve a local copy of the computed scalar (e.g., energy, property) via exatns_tensor_get_scalar. If one needs a slice of some computed tensor instead (e.g., density matrix), exatns_tensor_get_slice will return a local copy of the requested tensor slice. All created tensors need to be explicitly destroyed once no longer needed via exatns_tensor_destroy.

For the sake of completeness, let us briefly discuss the current parallelization algorithm used by ExaTENSOR for a distributed execution of tensor contractions on many GPU-accelerated HPC nodes. We should immediately note that the current algorithm is not communication optimal and also has other inefficiencies that we are currently addressing. In the current work, however, our goal was simply to extend the size of molecules that can be treated with relativistic coupled cluster theory via utilizing large-scale GPU-accelerated HPC platforms. The work on optimizing our parallel algorithms and their execution will follow in the near future.

The ExaTENSOR task-based parallel runtime consists of two types of MPI processes: managers and workers. Managers accept incoming tensor instructions (tensor operations, e.g., tensor contractions) and decompose them into smaller pieces (tasks), recursively. The tensor operation decomposition is induced by the decomposed structure of the participating tensors, determined during tensor creation. At the lowest level of task granularity, the generated elementary tensor operations are distributed across all workers (specialized MPI processes). The current task distribution algorithm is based on data affinity (tasks gravitate toward the workers owning the largest operand) and dynamic load balancing. The latter is replaced with static load balancing for tensor contractions with large output tensors due to the inefficiency of the MPI_Accumulate operation. Finally, the elementary tensor operations (tasks) received from managers are executed by workers, which includes allocation of memory resources, remote data prefetch or accumulation, and actual execution on either a multithreaded CPU or one or more GPUs in a fully asynchronous manner.

All computational aspects discussed above are taken care of by the ExaTENSOR parallel runtime and cannot be changed by the user of ExaCorr. Job-specific tuning and optimization of the parallelization are, however, possible by setting environment variables and/or specific keywords in the input. This provides control over the amount of memory used on a single node, whether GPUs are to be used, how OpenMP threads are distributed and mapped to CPU cores, etc.

### 3.4 Molecular Spinors: Interface to DIRAC and ReSpect

The ExaCorr module was designed with modularity in mind, so it would be easy to interface with other quantum–chemical packages. For convenience, we currently use the build infrastructure of DIRAC, but the code can also be compiled and used as a stand-alone program since the minor dependencies on some specific modules of DIRAC can be easily removed.

**Figure 2:**
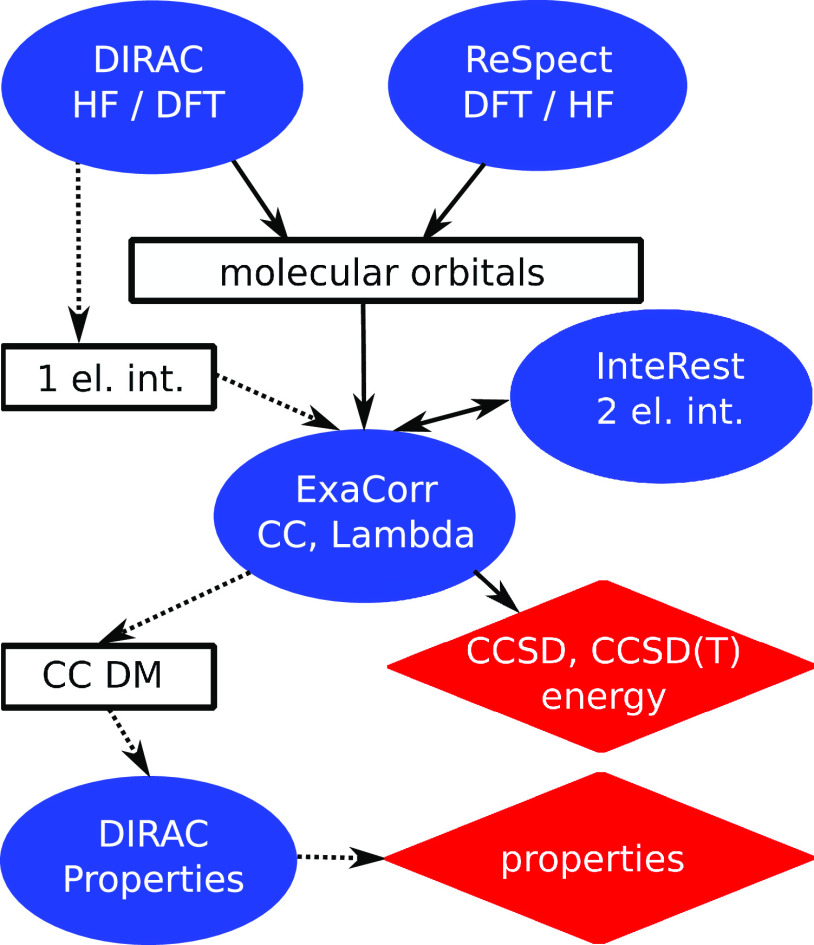
Workflow of ExaCorr computations; details can be found in the text.

ExaCorr requires two files with information to be present: a job input file and a file containing information about the molecular spinors. A complete diagram of the interface is depicted in Figure 2.

The input file (exacc.inp) contains the options controlling the coupled cluster computations and should at least include the definition of the active occupied and active virtual spinor spaces. Spinors outside this active space are considered as belonging to the frozen core (for the occupied spinors) or as deleted (for the virtual spinors). In the following, we will consider the occupied and virtual spaces as pertaining to these (potentially reduced) subspaces of the full spinor spaces defined in the molecular spinor file. Examples for additional options are convergence thresholds, choice of coupled cluster wave functions (CCD/CCSD/CC2/CCSD(T)), a switch to enable the computation of the density matrix, and several more technical keywords. A complete list can be found in the Supporting Information. These options can also be set in the DIRAC input (dirac.inp) if the ExaCorr module is called directly from DIRAC.

The second file can either be DIRAC’s molecular spinor file, DFCOEF, or the RSD_MOS file from ReSpect.^35^ These interface files contain three different sets of data defining the canonical molecular spinors: (i) information about the basis set, (ii) the coefficients of the molecular spinors, and (iii) the spinor energies thereof for the Fock matrix expression used in the generating SCF procedure. An optional input file (MRCONEE) containing one-electron integrals can be generated by the MOLTRA module in DIRAC. This additional data can be used to recompute the Fock operator for open-shell cases, for which the DIRAC definition,^54^ used to define the spinor energies, differs from the simple KU formalism assumed in ExaCorr. Results of the CC calculations are provided in the form of a text output file and an effective density matrix, in case the lambda equations are solved as well. This density matrix can be used by DIRAC to compute a wide range of molecular properties. As DIRAC assumes a KR formalism, the latter type of calculation is currently limited to Kramers symmetric (closed-shell) systems.

### 3.5 Index Transformation Algorithms

For relativistic calculations in which the size of the AO space is usually an order of magnitude larger than the MO -space, the transformation of the two-electron integrals from the atomic to the molecular basis can amount to a significant fraction of the overall computational expense. There are different approaches to implement these transformations differing in memory requirements and operation count. In ExaCorr, the current default is the standard Yoshimine^55^ scheme with *n*^5^ scaling, which reads for the Coulomb interaction in the X2C models as
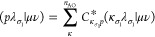
18
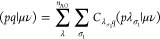
19
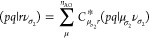
20
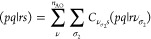
21where *p*, *q*, *r*, and *s* are the molecular spinors; κ, λ, μ, and ν are the spatial atomic orbitals; and σ_1_ and σ_2_ denote the spin for electrons 1 and 2, respectively. In this procedure, we make use of the fact that the AO spinors are defined as simple products of spatial and spin functions so that the spin integration reduces to additional summations in the second and fourth steps of transformation. The antisymmetrization and reordering, ⟨*pr*∥*qs*⟩ = (*pq*|*rs*) – (*ps*|*rq*), is done after the index transformation is completed. By making use of permutational symmetry, only six unique classes of molecular integrals are used, which can be generated on the basis of three classes of half-transformed integrals (vv, vo, and oo, where v and o stand for virtual and occupied spaces, respectively). The number of atomic orbitals (*n*_AO_) can become quite large, which makes it impractical to work with the complete atomic two-electron integral tensor (κλ|μν). The default in the current code is to slice the last index and work with a subspace thereof. This does not increase the operation count of the algorithm and is in practice sufficient to reduce the memory footprint due to handling of the AO integral tensor. This choice has the benefit of keeping large spaces for the other indices, making the tensor contractions optimally efficient.

### 3.6 Coupled Cluster Implementation

The general approach for applying the ExaTensor library is outlined in Figure 1. After reading and processing the input data, basis set information, spinor energies, MO coefficients and, optionally, the one-electron integrals are stored as global variables and broadcasted to all nodes using MPI. Three different spaces are defined, for the atomic orbitals, the occupied spinors, and the virtual spinors. In addition, the ExaCorr-specific methods are registered in the ExaTENSOR library interface. Apart from the already-mentioned ERI generation by InteRest, this, e.g., comprises methods to initialize a tensor with MO coefficients, initialize a tensor with one-electron integrals, scale a tensor with denominators, or project on a subspace. After these preliminary steps, the ExaTENSOR library is started and MO integrals are computed by transforming the ERIs to the MO basis. The MP2 amplitudes are subsequently computed to obtain an initial guess for the CC amplitudes. These CC amplitudes are then refined in an iterative procedure, the working equations for which can be found in the Supporting Information or ref (25). As the convergence of the nonlinear coupled cluster iterations can be slow, we have implemented the DIIS scheme^37^ and are also assessing the less memory-demanding CROP algorithm.^56^ In the current implementation, all necessary tensors are created before the iterative procedure starts, allowing for a priori assessment of the maximum memory footprint of the run.

Triple excitations require tensors of the size *n*_occ_^3^*n*_vir_^3^. For the full triples, these amplitudes need to be determined iteratively, which requires a significant amount of memory and number of operations. For the perturbative treatment of the triple excitations considered here, memory requirements can be reduced by splitting the occupied space and using three nested loops over these subspaces (of dimension *n*_red_) to evaluate all contributions. This results in a memory requirement of *n*_red_^3^*n*_vir_^3^ in addition to the memory required for the coupled cluster amplitudes, the two-electron integrals, and the Fock matrix. Permutational symmetry is used to speed up the computation by only computing unique blocks. The equations for the triples corrections are listed in the Supporting Information.

For the calculation of molecular properties, the equations for the Lagrange multipliers **λ** have to be solved, which can be done in much the same way as described above for the CC equations, including the use of DIIS to reduce the number of iterations. The **λ** and amplitude tensors are then combined according to the equations in the Supporting Information (see also ref (41)) to obtain the one-particle density matrix. In the case of the TAL-SH implementation, the tensor elements can be accessed directly and written to file. For ExaTENSOR, a local copy is first created in the form of a TAL-SH tensor, which is then written. The properties module in DIRAC can read these data and compute the properties.

## 4 Computational Details

All calculations were performed with development versions of the DIRAC^57^ and ExaCorr software package; details on the particular revisions used in the calculations described below are apparent in the respective output files that are provided within a separate repository.^46^ The geometries of the systems are also included in this collection.

The reference orbitals and single-determinant reference wave functions have mostly been obtained by the SCF implementation in DIRAC, which is a Kramers-restricted implementation. To enforce Kramers symmetry for systems that have an odd number of electrons or have near-degeneracies at the Fermi level, an average-of-configuration (AOC) approach is used in DIRAC.^54,57,58^ In contrast, the ReSpect code performs Kramers-unrestricted (KU) calculations, in which the Kramers symmetry is not imposed.^35^ For the utilization of spinors generated by the AOC procedure in DIRAC, some additional features are needed for the interface, as the definition of the Fock matrix in DIRAC differs from the KU Fock matrix, the definition assumed in ReSpect and ExaCorr. For closed-shell molecules, the difference between the AOC and KU Fock matrix expressions disappears and spinor energies can be read in from the DIRAC program and are sufficient to define the reference Hamiltonian. For open-shell molecules, one may either employ the ReSpect code or another code that has a compatible KU Fock matrix definition or recomputes the Fock matrix during the CC stage of the calculation. Both cases result in the use of a KU Hartree–Fock expression for a given reference determinant that is chosen by the user of the program. This is important for perturbation treatments, because the diagonal of the Fock matrix is then used to define the zeroth-order Hamiltonian.

Unless otherwise noted, we employed uncontracted Dyall basis sets of double (dyall.v2z), triple (dyall.v3z), or quadruple (dyall.v4z) zeta quality.^59−61^ The set of spinors included in the correlated calculations generally consists of a subset of the total set of spinors. By default, these are selected by energy thresholds corresponding to relatively high-lying occupied and low-lying virtuals, with energies between −10 and 20 hartree.

In the case of lanthanide monofluorides (LnFs) and the uranium hexafluorides (UF_6_) dimer, geometries were optimized at DFT level with the ADF^62^ code using the scalar-relativistic ZORA Hamiltonian,^63^ the Perdew–Burke–Ernzerhof (PBE) functional,^64^ and triple zeta basis sets with one polarization function (TZP).

Further, molecule-specific, computational details are listed below.

### 4.1 Lanthanide Monofluorides

For LaF and YbF, the AOC-SCF approach was applied using DIRAC either employing X2C-AMFI^30,32^ or the one-component nonrelativistic (NR) Hamiltonian. In the case of EuF, KU calculations were performed with ReSpect^35^ using the one-component nonrelativistic or X2C-1e Hamiltonian.^31^ Several thresholds for the occupied and virtual spinors were considered for the double zeta basis set, and the same values were employed for larger basis sets.

### 4.2 Argon Binding to Gold

For argon atoms bound to gold clusters, we employed the X2Cmmf Hamiltonian,^18^ with the structures being taken from ref (65). The default energy thresholds in the coupled cluster step have been employed. The numbers of correlated electrons for the systems considered are 46 (AuAr^+^), 60 (AuAr_2_^+^), 74 (AuAr_3_^+^), 88 (AuAr_4_^+^), and 102 (AuAr_5_^+^).

### 4.3 Uranium Hexafluoride Dimer

In the case of UF_6_ and (UF_6_)_2_ calculations, the X2Cmmf Hamiltonian^30,31^ was applied, except for some smaller scaling investigations for which we used X2C-AMFI.^30,32^ In these smaller computations, the cc-pVDZ and dyall.v2z basis sets were selected for F and U, respectively. The larger, more accurate computations employed the corresponding triple zeta basis sets. The energy thresholds for the included spinors were −35 and 80 hartree for smaller computations and −10 and 8 hartree for the triple zeta ones.

In the computations, the distance between uranium and fluorine in a monomer was fixed to an experimental value of 1.996 Å.^66^ A restricted optimization using these monomers was performed for (UF_6_)_2_, and the structures were applied in the coupled cluster computations. In this case, we added the dispersion correction by Grimme to the PBE functional. Additionally, an optimization without restrictions was performed for the UF_6_ dimer at the DFT level to estimate the U–F bond distances for this level of theory.

### 4.4 Uranyl Tris-nitrate Complex

Calculations of the *zz* component of electric field gradient (EFG) at the U nucleus (*q*_*zz*_) for the uranyl tris-nitrate ([UO_2_(NO_3_)_3_]^−^) complex have been performed at the X-ray structure for the RbUO_2_(NO_3_)_3_ crystyal,^67^ employing the X2C-AMFI^30,32^ and taking into account the picture change of the EFG operator. In addition to CC, we have performed DFT calculations with the B3LYP, PBE0, and CAMB3LYP density functionals.

For the property CC calculations, we considered occupied spinors with energies higher than or equal to (a) −6 hartree (106 electrons, in which the U 5d is correlated as done for other uranyl complexes^68^), (b) −22 hartree (156 electrons, in which the U 4f and all electrons for the light atoms are correlated), and (c) −4500 hartree, which amounted to correlating all 202 electrons. These three occupied spaces are combined with virtual spinors with energies up to and including (a) 5 hartree for both double and triple bases (543 and 649 virtuals, respectively); for only double zeta bases, (b) 20 hartree (680 virtuals), (c) 50 hartree (818 virtuals), (d) 150 hartree (896 virtuals); and for triple zeta bases, (e) 7 hartree (944 virtuals). The total numbers of virtual spinors are 1286 and 2076 for double and triple zeta bases, respectively. We have not performed CCSD calculations with quadruple zeta bases.

## 5 Results and Discussion

Our first goal was to verify the correctness of the new implementation. To do so, we compared the results of the new implementation using TAL-SH or ExaTENSOR to the results obtained by the RELCCSD implementation in DIRAC.^22,57,58^ Comparisons of the energies for H_2_O, LiO, and CuAr_*n*_^+^ are in the Supporting Information. To check the property implementation, we compared the dipole moment, EFG, and the nuclear quadrupole coupling constant (NQCC) of CHFClBr and UF_6_ for different implementations, which can also be found in the Supporting Information; the output files are provided in a separate repository.^46^

A few systems were selected to show the capabilities of the new implementation in the investigation of heavy-element systems. First, we consider the ionization energies of three lanthanide monofluorides (LnF) species, LaF, YbF, and EuF since, from a methodological perspective, these calculations allow us to demonstrate the usage of our implementation for both closed- and open-shell configurations. Second, the binding of argon atoms to gold cations has been studied including triples corrections, which are necessary to achieve chemical accuracy. In the subsequent section, results for uranium hexafluoride and its dimer are presented as well as some information about the scaling of the new code. Finally, the electric field gradient of the uranyl tris-nitrate complex was computed as an example for evaluation of electronic properties in a larger molecule.

### 5.1 Ionization Energies of Lanthanide Monofluorides

Lanthanides are often treated using density functional theory, but results are shown to have a strong dependence on the exchange–correlation functional that is selected.^69^ Coupled cluster theory can provide more accurate and precise results and has been applied in conjunction with more approximate methods to account for relativistic effects, like the one-component Douglas–Kroll Hamiltonian^70^ and effective core potentials.^71^ The current implementation, and in particular the interface for the ReSpect code, provides a way to investigate the (generally open-shell) ground states for such systems with full inclusion of relativistic and core correlation effects in coupled cluster theory.

Before proceeding to the discussion of our results for the ionization energies themselves, we shall discuss the requirements, in terms of the number of occupied and virtual spinors necessary for obtaining reliable results. For this, we have decided to consider two sets of equilibrium structures, one for the neutral and the other for the ionized species. Our structures, obtained at the DFT level using the PBE functional, are shown in Table 1, together with experimental values and prior theoretical values. For these systems, DFT produces the experimentally observed trend, with EuF having the largest bond distance and YbF the smallest. As expected, there are some deviations as well, with the DFT bond distances being smaller than the experimental values for EuF and YbF but slightly larger for LaF.

**Table 1:** Experimental Reference Values and Structures Used in the Computations for Lanthanide Monofluorides and DFT Bond Distances Applied in the Computations

	*r*_e_ (exp.)	*r*_e_ (PBE)	*r*_e_ (cation, PBE)	*r*_e_ (ECP, CCSD(T))^71^
LaF	2.0234^72^	2.0293	2.0150	2.0215
EuF	2.083^73^	2.0676	1.9992	2.0750
YbF	2.016516^74^	1.9868	1.9345	2.0204

Now, concerning the coupled cluster calculations themselves, we first investigated the convergence of the energies with the number of active occupied and virtual spinors. The reason for such an investigation is that employing the complete set of virtual spinors is typically not needed in relativistic calculations of heavy elements. This is due to the use of uncontracted basis sets, which leads to a significant number of virtual spinors being mostly located in the chemically inactive core region. These types of spinors can be deleted without affecting the results much. In the current work, we identify such spinors by a simple energy criterium, relying on the observation that the large kinetic energy of these solutions puts them in the upper range of energies obtained by Fock matrix diagonalization. More advanced schemes, such as use of approximate natural orbitals are also possible and under development. Regarding the choice of occupied spinors to be included, one needs to take into account that for lanthanides, the closeness (in radial extent) of the open-shell 4f and other electrons that would otherwise be considered as core (4s–4d) may require that they are correlated alongside the (5s, 5p) valence.

We present in Table 2 the results of such an investigation for the YbF, which had previously been investigated by some of us^61^ and which was found to be particularly sensitive to electron correlation treatment. We provide equivalent tables for LaF and EuF as the Supporting Information due to the fact that these exhibit the same trends as discussed below.

**Table 2:** Ionization Potential in eV of YbF for Different Numbers of Correlated Spinors Employing the dyall.v2z Basis Set^a^

threshold_low_	threshold_high_	nocc	nvir	% occ	% vir	CCSD
–20	2.3	49	89	63	21	4.49
–20	6	49	137	63	32	5.89
–20	150	49	267	63	63	5.89
–20	10 000	49	367	63	86	5.89
–60	10	61	155	78	36	5.90
–60	20	61	195	78	50	5.90
–60	150	61	267	78	63	5.90
–3	40	31	213	40	50	5.95
–20	40	49	213	63	50	5.90
–40	40	51	213	65	50	5.90
–60	40	61	213	78	50	5.90
–400	40	77	213	99	50	5.90
exp						5.91 ± 0.05^75^

a The number of occupied and virtual spinors refers to the neutral molecule; for the cation, one of these occupied spinors becomes a virtual spinor. The ΔSCF ionization potential computed using the reference determinant wave functions was 5.48 eV. The spinor thresholds are listed in atomic units.

From Table 2, we can identify two main trends: (a) Employing a too small virtual space (comprising around 21% of the total number of virtuals), even with a fairly large number of occupied, yields a (strong) underestimation of the ionization energy at the CCSD level (−1.41 eV). A modest increase in the number of virtuals (including around 30% of the virtuals) greatly reduces this underestimation and brings values closer to the experimental value. Further increases in the number of virtuals past 60% yield no significant difference in the CCSD ionization energies. (b) Employing a converged virtual space (>30%) but not enough occupied spinors overestimates the ionization energies, though not by much (around +0.05 eV). Possible choices for the occupied space are to correlate only F(2s^2^2p^6^) and Yb(5s^2^5p^6^4f^14^) (comprising 40% of the occupied space) or to include the Yb(4s^2^4p^6^4d^10^) shells as well (63% of the occupied space).

For reliable results, we find that the following electrons need to be correlated: La(4s^2^4p^6^5s^2^4d^10^5p^6^), Eu(3s^2^3p^6^4s^2^3d^10^4p^6^4d^10^5s^2^5p^6^6s^1^4f^7^), and Yb(4s^2^3d^10^4p^6^5s^2^4d^10^5p^6^6s^1^4f^14^), which can be achieved by employing energy thresholds of −20, −200, and −60 hartree for LaF, EuF, and YbF, respectively. The 2s^2^ and 2p^6^ of F are always included, and the 1s^2^ is omitted for LaF. In the case of Yb, the neutral molecule is an open-shell system, while the cation only has closed shells. The opposite is true for La. EuF was considered as an example with a high-spin state; the neutral molecule as well as the cation has several open shells.

Following our analysis of what are the minimum requirements in terms of occupied and virtual spinors for obtaining converged ionization energies, we investigate the adiabatic and vertical ionization potentials for the three molecules, computed for the basis set of increased quality, for two classes of Hamiltonians (nonrelativistic and X2C). The values are listed in Table 3.

**Table 3:** Vertical (a) and Adiabatic (b) Ionization Energies (in eV) for the Lanthanide Monofluorides^a^

			ΔSCF		CCSD			CCSD+T		CCSD(T)		CCSD-T	
		basis	(a)	(b)	(a)	(b)	T1	(a)	(b)	(a)	(b)	(a)	(b)
LaF	NR	2z	3.41	3.44	5.03	5.06	0.01	5.15	5.18	5.11	5.14	5.10	5.13
		3z	3.43	3.44	4.71	4.71	0.02	4.90	4.91	4.84	4.84	4.82	4.74
		4z	3.43	3.43	4.61	4.60	0.02	4.81	4.81	4.74	4.74	4.72	4.72
		∞z	3.42	3.43	4.53	4.53		4.74	4.74	4.66	4.66	4.65	4.64
	X2C	2z	4.93	4.96	5.91	5.93	0.01	5.96	5.99	5.96	5.98	5.96	5.98
		3z	4.87	4.87	5.87	5.87	0.01	5.98	5.98	5.96	5.96	5.96	5.95
		4z	4.86	4.86	5.87	5.86	0.01	5.99	5.99	5.97	5.97	5.97	5.96
		∞z	4.85	4.84	5.87	5.86		6.00	5.99	5.98	5.97	5.97	5.96
	exp.		6.3 ± 0.3^76^										
EuF	NR	2z	4.73	4.75	5.08	5.12	0.01	5.10	5.15	5.10	5.15	5.11	5.15
		3z	4.72	4.74	5.10	5.13	0.01	5.13	5.17	5.13	5.17	5.13	5.17
	X2C	2z	5.04	5.07	5.46	5.51	0.01	5.51	5.57	5.51	5.56	5.51	5.56
		3z	5.02	5.05	5.48	5.52	0.01	5.54	5.58	5.53	5.57	5.53	5.57
	exp.		5.9 ± 0.3^77^										
YbF	NR	2z	5.04	5.09	5.39	5.39	0.01	5.43	5.43	5.42	5.43	5.42	5.43
		3z	4.93	4.98	5.40	5.43	0.01	5.47	5.49	5.45	5.47	5.46	5.48
		4z	4.93	4.98	5.42	5.44	0.01	5.59	5.60	5.56	5.57	5.57	5.58
		∞z	4.93	4.98	5.44	5.45		5.67	5.68	5.64	5.65	5.65	5.66
	X2C	2z	5.48	5.49	5.90	5.87	0.05	6.51	6.44	5.57	5.56	5.67	5.65
		3z	5.44	5.46	6.00	5.98	0.09	7.76	7.73	5.30	5.29	5.76	5.75
		4z	5.44	5.46	6.00	5.97	0.05	6.82	6.78	5.57	5.56	5.75	5.74
		∞z	5.44	5.46	6.00	5.97		6.13	6.08	5.77	5.75	5.75	5.73
	exp.		5.91 ± 0.05^75^										

a The active ranges for LaF, EuF, and YbF are −20 to 40, −200 to 200, and −60 to 40 hartree, respecitvely. The X2C Hamiltonians used were X2C-AMFI for LaF and YbF (spinors obtained with the DIRAC code) and X2C-1e for EuF (spinors obtained with the ReSpect code). The complete basis set limit values (∞z) have been obtained with a two-point extrapolation formula based on the 3z and 4z values.

The largest change of the ionization potential is due to the inclusion of relativistic contributions. Regarding LaF, the ionization potential is larger by about 1.3 eV (CCSD) or 1.4 eV (SCF) for the X2C-1e Hamiltonian than that for the nonrelativistic one. For EuF/YbF, the changes are somewhat smaller; increases of about 0.4/0.5 and 0.3/0.4 eV were obtained for the coupled cluster and ΔSCF, respectively.

The inclusion of electron correlation by CCSD results in an increase of the ionization potential by about 1, 0.4, and 0.5 eV for LaF, EuF, and YbF, respectively. These are the values for the X2C-1e Hamiltonian; the changes in the nonrelativistic case are similar. The perturbative triples corrections are the smallest for EuF, probably due to the relatively simple high-spin ground states of both the neutral and the cation, which can be well described by the Kramers-unrestricted reference wave functions obtained with ReSpect. They increase the ionization potential by less than 0.06 eV. The triples add about 0.1 eV in the case of LaF for X2C-1e and slightly more for the nonrelativistic Hamiltonian. While the triples in the nonrelativistic case are similar (between 0.1 and 0.2 eV) for YbF, the values for X2C are much larger. The fourth-order correction increases the IP by about 1.7 eV, the fifth-order correction results in values about 0.7 eV below the CCSD ones. A similar observation has been reported in ref (61). These large values are an indication that a perturbative inclusion of triples is insufficient, in agreement with the large *T*_1_ values, see Table 3. This is probably caused by a mixing of excited states with closed and open f-shells, which was observed to cause a large change in ground-state polarizability of the Yb atom^78^ and a change in the nuclear quadrupole coupling constant in ref (79).

For an increase of the basis set size, the ionization potential of the reference determinant becomes smaller except for the vertical transition of LaF using the nonrelativistic Hamiltonian. Going from double to triple zeta basis sets, the coupled cluster ionization potential increases for YbF and EuF, although the changes are below 0.03 eV in the latter case. Regarding LaF, a decrease of the ionization potential is observed at the coupled cluster level.

The adiabatic IP should be smaller than the vertical one if the equilibrium distances are correct. For the HF reference, this is never the case as the electron correlation is missing in contrast to the DFT that was used to determine the bond distances. In the case of the coupled cluster, the correct order of the vertical and adiabatic IP is obtained for the X2C-1e Hamiltonian and triple or quadrupole zeta basis set, indicating that the DFT bond distances are close. EuF always shows the wrong order, probably because the bond distances are not accurate enough, which is probably also reflected in the larger differences between the theoretical and experimental values in Table 1.

The best estimates from Table 3 are 5.97, 5.57, and 5.97 eV for LaF, EuF, and YbF, respectively, while values of 6.3, 5.9, and 5.91 eV were determined in experiments (Table 1). One of the reasons for these discrepancies is the large experimental uncertainties (especially for LaF and EuF), while also the neglect of zero point energies in our values will play a role. Considering these sources of errors, the energies show an acceptable agreement.

Systems with open shells, like treated above, can be difficult to describe using coupled cluster since CC is based on a single reference determinant. The t1 amplitudes recover a portion of the static correlation, which makes treatment possible when there is one dominant determinant, but in cases with several important configurations, multireference methods are necessary.^80^ A related difficulty appearing in a two-component treatment is that the spinors are no longer eigenfunctions of  At the SCF level, this can be handled by an average-of-configuration approach,^54^ which occupies all relevant configurations, resulting in spinors with varying spin-up and spin-down contributions. Making a proper selection of such spinors to form a single-determinant reference wave is, however, difficult in the general case. The exception is cases with only a single unpaired electron in which either spinor of the singly occupied Kramers pair can be taken to construct the reference determinant. In the current work, molecules were selected that are still rather easy to treat. YbF and LaF have only one unpaired electron and thus belong to this important special case of simple open-shell molecules. For neutral and positively charged EuF, one determinant can qualitatively correctly describe the ground states if we allow for a KU spinor optimization that is able to converge to a “high-spin” state. This is possible with ReSpect.

While the use of Kramers restriction in an averaged SCF is feasible for simple open-shell systems, it does lead to an inconsistency in the definition of the reference Fock operator and orbital energies between the KR SCF program and the KU CC implementation. This is formally not a problem as our working equations do not require the use of a diagonal Fock operator but make working with denominators consisting of spinor energy differences between occupied and virtual spinors more complicated. With unmodified orbital energies, the energy difference between the highest occupied spinor (one of the two open-shell spinors) and the lowest unoccupied spinor (its Kramers partner) would be zero. There are several ways to deal with this complication. One is to recompute the spinor energies according to the KU Fock matrix expression. This will induce an energy gap and make it possible to apply denominators. This simple approach was applied to the results for LaF and YbF presented in Table 3.

### 5.2 Binding Energies of Argon Atoms to Gold Atoms

Gold is one of the most nonreactive metals in the periodic table, and noble gases are also exceptionally inert. Nevertheless, the AuNe^+^ dimer was reported in 1977^81^ and early computations suggested a covalent bond between gold cations and noble gas atoms,^82^ which is supported by recent experimental results.^83^ A theoretical study observed strengthening of the binding in small gold clusters if noble gas atoms are attached.^84^ This covalency is in part attributed to the relativistic nature of the heavy Au; this makes it necessary to include these contributions in theoretical studies. Recently, the significant influence of argon atoms on the IR spectra and bonding of small gold clusters was observed.^85^ Here, we want to compute the interaction of a single gold atom with argon using the reliable coupled cluster method in combination with the X2Cmmf Hamiltonian. A summary of the current state of research on noble gas-noble metal compounds can be found in a recent review.^86^

First, the energy of the AuAr dimer was computed for different internuclear distances. The equilibrium bond distances for the AuAr dimer were determined by fitting a Morse potential to about 5 points. For the dyall.v4z basis set, equilibrium bond distances of 2.50, and 2.47 Å were obtained by CCSD and CCSD(T), respectively. The MP2 value is about 0.1 Å smaller than the CCSD one, and the HF value is about 0.3 Å larger. The triples correction reduces the equilibrium distance by about 0.03 Å, a detailed table is in the Supporting Information. This general trend is observed for all basis sets, while the bond distance is about 0.05 smaller for 3z than for 2z. The structures of the larger systems were obtained by density functional theory.^65^ Coupled cluster binding energies are listed in Table 4. There is a strong dependence on the method; Hartree–Fock underestimates the CCSD binding energies by 0.4–1.5 eV, MP2 overestimates them by 0.04–0.4 eV. The triples correction are also significant; they increase the binding energy by 0.16/0.20 eV for the fourth-order +T and 0.14/0.18 for the fifth-order (T)/–T considering the dyall.v2z/dyall.v3z basis set, excluding the AuAr dimer with smaller triples contributions. The dimer constitutes a special case as the structures were optimized at the coupled cluster level. For this reason, significant HF binding energies were obtained as they are computed for longer bond distances as the CC ones. If the basis set is increased or extrapolated, the CCSD energy increases by about 0.04 eV, except for the dimer with smaller changes. The growth of the CCSD+T/(T)/-T energy is about 0.08 eV in going from the double to triple zeta basis set, excluding the AuAr dimer. The energy per argon atom reaches its maximum for AuAr_2_^+^ with about 0.78 eV at the CBS CCSD level of theory. For AuAr_4_^+^, two structures have been computed to assess the relative stability of a planar and a 3D arrangement. Independent of the basis set and method, the three-dimensional structures are found to be lower in energy.

**Table 4:** Total Binding Energy (Δ*E*, in eV) of AuAr_*n*_ Systems with dyall Basis Sets of Different Cardinal Numbers^a^

			Δ*E*					
system	basis	V	HF	MP2	CCSD	CCSD + T	CCSD(T)	CCSD – T
AuAr^+^	2z	136	–0.1341	–0.5260	–0.4620	–0.5267	–0.5165	–0.5156
	3z	230	–0.1401	–0.5993	–0.4699	–0.5519	–0.5408	–0.5400
	4z	400	–0.1456	–0.6408	–0.4817	–0.5689	–0.5586	–0.5581
	∞z		–0.1496	–0.6711	–0.4904	–0.5814	–0.5716	–0.5713
AuAr_2_^+^	2z	168	0.0845	–1.2292	–1.0356	–1.1981	–1.1723	–1.1706
	3z	288	–0.0219	–1.4124	–1.0890	–1.2813	–1.2563	–1.2550
	4z	508	–0.0399	–1.4938	–1.1161			
	∞z		–0.0531	–1.5532	–1.1360			
AuAr_3_^+^	2z	200	–0.0140	–1.3416	–1.1491	–1.3100	–1.2875	–1.2860
	3z	346	–0.0816	–1.5202	–1.1899	–1.3902	–1.3669	–1.3652
	4z	616	–0.1019	–1.6124	–1.2268			
	∞z		–0.1167	–1.6797	–1.2538			
AuAr_4_^+^(3D)	2z	232	–0.1061	–1.4673	–1.2681	–1.4267	–1.4090	–1.4078
	3z	404	–0.1367	–1.6474	–1.3009	–1.5092	–1.4890	–1.4872
	4z	724	–0.1590	–1.7527	–1.3491			
	∞z		–0.1753	–1.8295	–1.3843			
AuAr_4_^+^(2D)	2z	232	–0.1605	–1.4326	–1.2530	–1.4024	–1.3863	–1.3852
	3z	404	–0.1783	–1.5967	–1.2769	–1.4750	–1.4563	–1.4545
	4z	724	–0.2001	–1.6978	–1.3243			
	∞z		–0.2160	–1.7715	–1.3588			
AuAr_5_^+^	2z	264	–0.2217	–1.5945	–1.3898	–1.5398	–1.5301	–1.5750
	3z	462	–0.2059	–1.7730	–1.4126	–1.6238	–1.6100	–1.6081

a In all cases, spinors with energies between −10 and 20 hartree have been included in the correlation treatment. The number of virtual spinors in each case (V) is shown; see Section 4 for the number of correlated electrons for each species. The complete basis set limit values (∞z) have been obtained with a two-point extrapolation formula based on the 3z and 4z values.

These preliminary findings will be incorporated in a larger investigation of Ar bound to gold clusters in conjunction with infrared multiphoton dissociation experiments.^87,88^ For such investigations, it is essential to be able to have reliable benchmarks of DFT calculations, which will become possible with this new implementation,

For these systems, it is possible to compare the performance of the new implementation to the RELCCSD code in DIRAC.^22,57,58^ In Table 5, the timings for the three parts of the coupled cluster computation are listed, the integral transformation, the CCSD iterations, and the computation of the perturbative triples. The new ExaCorr implementation is always faster than the RELCCSD implementation without symmetry. Including the symmetry significantly reduces the computational time, especially for systems with linear symmetry (AuAr^+^, AuAr_2_^+^). In these systems, the integral transformation of RELCCSD with symmetry takes about the same time as in ExaCorr, while the CCSD iterations take less time. However, once the system has less symmetry (AuAr_3_^+^, Cs), ExaCorr is significantly faster than RELCCSD for which calculation of the triples correction turned out to be infeasible for this system.

**Table 5:** Wall Time in Seconds for the New Implementation and the RELCCSD Release 2019 Reference Implementation, with Symmetry (R19s) and without Using Symmetry (R19)^a^

step	system	nodes	R19	R19s	ExaCorr
integrals	AuAr^+^	4	371	71	106
	AuAr^+^	8	308	70	58
	AuAr_2_^+^	8	715	87	77
	AuAr_3_^+^	8	1602	810	133
	AuAr_3_^+^	20	1450	703	87
CCSD	AuAr^+^	4	327	7	84
	AuAr^+^	8	232	6	71
	AuAr_2_^+^	8	1129	7	73
	AuAr_3_^+^	8	2512	849	183
	AuAr_3_^+^	20	1585	586	143
triples	AuAr^+^	4	2055	42	1555
	AuAr^+^	8	3130	46	1294
	AuAr_2_^+^	8	>5200	90	2666
	AuAr_3_^+^	20	>5200	>5200	5821

a All computations were performed using the dyall.v2z basis set. There are 243/42 atomic orbitals, 32/14 occupied and 104/32 virtual spinors per gold/argon atom.

### 5.3 Binding Energy of Uranium Hexafluoride Dimers

Uranium hexafluoride is used in the gaseous form in enrichment methods for nuclear fuels. To simulate the behavior of this gas under different conditions, an accurate description of the intermolecular interaction potential is important. Early attempts to describe the interaction of molecules were based on potentials derived from thermophysical data and spectroscopy.^89,90^ In quantum chemistry, the properties^91−94^ and reaction pathways^95^ of the monomer have been mainly studied employing relativistic DFT. To describe the interaction of two such units, it is important to account for relativistic^91^ as well as dispersion effects accurately. As the electronic structure of the dimer is not problematic and well described by a single reference determinant, coupled cluster theory can be used to provide accurate reference data. Since the computations are rather expensive, due to the number of electrons that needs to be correlated, this particular system is well suited for testing our implementation.

First, we performed computations for the UF_6_ monomer on different numbers of nodes. Tables 6 and 7 display the obtained timings for the double and triple zeta basis set, respectively. As evident from Table 6, our code scales up to 48 nodes for such a small system before the ExaTENSOR worker processes begin to starve and/or have load balancing issues. Naively, the total number of tasks as well as load balancing could in principle be improved with finer task granularity, which can be achieved by decreasing the dimension segment size from 75 to 50, but the increased inter- and intranode communication overheads then in fact lead to an overall increase of the computational time, as can be seen in Table 6. The better performance and scaling of large tensor contractions enhance the difference between the CCD and CCSD formalisms. While the additional tensor contractions related to the inclusion of single excitations are at most of order *n*^5^, CCSD iterations took noticeably more time than the CCD ones as these additional contractions were computationally less efficient at the time of experimentation due to poor load balancing. Since then, the efficiency of the singles tensor contractions has been improved via a more even work distribution. The lambda equation iterations are slightly faster than the CCSD ones but otherwise behave similarly in terms of scaling. As the size of the AO basis is much larger than that of the MO basis, in particular, the first stages of the integral transformation can make up a large portion of the computational time. This is more important for larger AO sets. In Table 7, one may notice that for the smallest node count, this step even dominates the calculation. Therefore, index transformations require special attention and will be the first target for improvements using techniques like Cholesky decomposition that allow for reduction of the operation count without impacting the accuracy.

**Table 6:** Time in Seconds for Integral Transformation (*t*_I_) and Solving the Coupled Cluster *(t*_CCD_, *t*_CCSD_) and Λ Equations (*t*_Λ_) for UF_6_ Using the dyall.v2z Basis Set^a^

*n*	*t*_I_ (75)	*t*_I_ (50)	*t*_CCD_ (75)	*t*_CCD_ (50)	*t*_CCSD_ (75)	*t*_Λ_ (75)
16	841	1179	843	1314	2046	
24	654	902	711	1045	1869	1865
32	505	764	617	925	1645	1649
48	413	686	594	861	1512	1493
64	375	634	600	810	1545	1412

a The CCD, CCSD, and Λ equations, took resp. 10, 20, and 21 iterations to solve. For the selected thresholds of −35 to 80 hartree, 110 occupied and 474 virtual spinors are included. *n* is the number of Summit nodes. The number in parentheses in the header is the segment size used by ExaTENSOR for chunking the occupied and virtual vector spaces.

**Table 7:** Time in Seconds for Integral Transformation (*t*_I_) and Coupled Cluster (*t*_CCSD_) for UF_6_ Applying the dyall.v3z Basis Set^a^

*n*	*t*_I_ (75)	*t*_CCSD_ (75)
32	1685	1279
64	1191	1205
96	817	1081
128	687	973

a The CCSD equations took 21 iterations to solve. For thresholds of −10 to 8 hartree,l 70 occupied and 554 virtual spinors are active. *n* is the number of Summit nodes. The number in parentheses in the header is the segment size used by ExaTENSOR for chunking the occupied and virtual vector spaces.

Currently, the scalability and efficiency of our GPU-accelerated implementation is hindered by a number of factors. First, in order to amortize the cost of the Host-to-Device memory transfers, we have to maintain a large granularity of the stored tensor blocks. Although it is beneficial for absolute efficiency within a node, it can also cause work starvation at larger node counts when the system is relatively small. Second, for tensor contractions with a large output tensor (e.g., any four-dimensional output tensor), the static load balancing mechanism is activated due to the inefficiency of MPI_Accumulate operation required in the dynamic load balancing mechanism (the latter is now used for tensor contractions with a small output tensor). The static load balancing mechanism is based on the task-binding affinity dictated by the output tensor, which can lead to a noticeable load imbalance when the total number of the output tensor blocks is not divided evenly by the number of the worker processes. Finally, the third and the most important issue we observe is the serialization overhead caused by the generation of the global task list done by the manager processes. In particular, all runs reported in this work utilized only a single manager process. For the molecular systems reported here, the generation of the global task list could take a noticeable time as compared to the actual task execution time by all worker processes. This explains the low scalability slope observed in Tables 6 and 7, resulting in low parallel efficiency. We are currently addressing this issue by switching to multimanager configurations. Finally, we also observed occasional synchronization overheads for MPI-3 one-sided communications.

**Figure 3:**
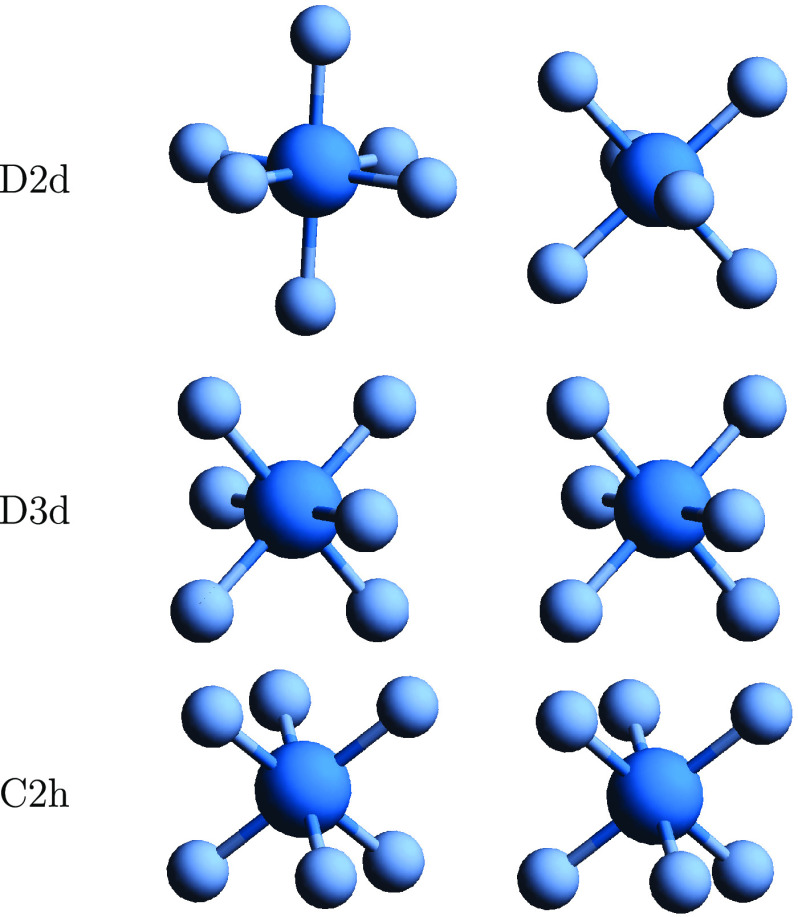
Orientation of the UF_6_ dimers.

To further assess performance on larger node counts, a larger system with a higher operation count is necessary. We therefore also investigated the (UF_6_)_2_ dimer to have a case with approximately 64 times more floating point operations to process. This system has not yet been treated at the coupled cluster level of theory, but a dimer interaction potential was computed with DFT,^96^ including relativistic effects via a relativistic effective core potential. Concerning the relative position and alignment of the two UF_6_ monomers, there are three minima that are depicted in Figure 3. They are designated by the symmetry of the complex. The energies and U–U bond distances are listed in Table 8. For the D2d complex, the smallest U–U distance and the highest binding energy were obtained. The C2h complex had the largest separation of the uranium atoms in the equilibrium, and for D3d, the smallest binding energies were obtained. The trend of the energies is the same for the different levels of theory, but the absolute values vary strongly. In the case of Hartree–Fock, the complexes are barely bound, while MP2 overestimates the binding energy and the CCSD and DFT values are rather close ranging from 0.13 to 0.19 eV.

**Table 8:** UF_6_ Dimer Bonding Energies (d*E*, eV) for a System with 140 Occupied and 1108 Virtual Spinors for Different Computational Methods^a^

		d*E*						
sym	U–U	DFT	HF	MP2	CCSD	*n*	*t*_I_	*t*_CC_/it.
D3d	5.144	–0.136	0.037	–0.154	–0.131	385	9244	687
D2d	5.139	–0.160	–0.003	–0.189	–0.178	513	8641	758
C2h	5.290	–0.150	–0.001	–0.163	–0.151	1025	8356	665

a The U–F distances have been fixed at 1.996 Å;^66^ the U–U distances are optimized with DFT and are listed in the first column in Å. *n* is the number of Summit nodes.

In the v3z computation of the dimer, 140 active occupied and 1108 active virtual spinors are taken into account in the coupled cluster computation. With a chosen segment size of 70 for both spaces, the *t*_2_ tensor consists of 1024 blocks. This means that we reach the principal scaling limit at 1024 MPI processes or, equivalently, 512 Summit nodes. If one compares the timings for runs with 385 and 513 nodes in Table 8, one can see a speedup for the integral transformation, but not for the coupled cluster iterations. The time dominating tensor contractions in the integral transformation are those in which indices are transformed from the atomic orbital basis to the virtual space, and these contain a relatively large number of tensor blocks to parallelize over, whereas in the coupled cluster iterations, most tensors are smaller than *t*_2_. Due to the necessity of avoiding remote accumulates and maintaining large granularity of tensor blocks for GPU processing, the coupled cluster workload simply does not have enough work items to efficiently parallelize over more than 385 nodes (for this specific molecular system). On the other hand, due to high memory demands, we could not use fewer nodes for this particular calculation. Such a situation will be characteristic for molecules with a large virtual-to-occupied spinor ratio, like our current UF_6_ dimer system with a significantly reduced occupied space (letting the occupied space include all electrons would restore the scaling to higher node counts). Currently, we are working on improving our original algorithm to address this issue.

Finally, to conclude our performance analysis, Tables 9 and 10 show the scalability of a few representative individual tensor contractions with an increased number of occupied spinors (280) that were performed after some very recent improvements in the ExaTENSOR library. In contrast to previous calculations, these runs were performed with multiple ExaTENSOR manager processes instead of a single manager process, and they dynamically switched between static and dynamic load balancing, as described in Section 3.3. Contraction 1 is a representative contraction of the last stage of the integral transformation: *V*(*v*,*v*,*v*,*v*)+ = *C*(*v*,*a*)*H(*v*,*v*,*v*,*a*), where *v* designates a virtual index placeholder and a designates an atomic index placeholder. Contraction 2 is a representative of a singles projection contraction: *Z*(*v*,*o*)+ = *V*(*v*,*v*,*o*,*v*)*T(*v*,*v*,*o*,*o*), where o designates an occupied index placeholder. Contraction 3 is the cost-dominating term in the doubles projection: *Z*(*v*,*v*,*o*,*o*)+ = *V*(*v*,*v*,*v*,*v*)*T(*v*,*v*,*o*,*o*). As one can see from Table 9, the parallel efficiency of Contraction 1 is not improved as compared to the overall parallel efficiency of the integral transformation reported in Table 7. However, the parallel efficiency of Contraction 2 and Contraction 3, which are two representative contractions from the coupled cluster singles and doubles equations, is much better than that of the original full CCSD run reported in Table 7. Indeed, for Contraction 2, we observe around 68% parallel efficiency from 32 to 128 nodes. For Contraction 3, we similarly observe around 67% parallel efficiency from 32 to 128 nodes, which is encouraging. Unfortunately, due to a shortage in computational time and some technical issues, we have not yet had a chance to rerun the full CCSD iterations with an updated algorithm, which will be done in the near future. For the sake of completeness, we have also run Contraction 3 with an increased number of virtual spinors (1120) on 512 and 1024 worker nodes, observing the execution times of 504.96 and 365.08 s, respectively. Although this confirms the scalability of larger tensor contractions to larger node counts, the corresponding parallel efficiency in the weak scaling regime is only 39%, which indicates communication overhead among other inefficiencies described above. At last, let us report the absolute efficiency of the cost-dominating Contraction 3, which is 44 and 30% on 32 and 128 nodes in Table 9, respectively, and 15% on 512 nodes in Table 10, which includes all reported inefficiencies.

**Table 9:** Strong Scaling Benchmark: Times in Seconds of Three Representative Tensor Contractions with 560 AO, 560 Virtual, and 280 Occupied Spinors^a^

no. of nodes	contraction 1	contraction 2	contraction 3
32	35.70	11.82	96.68
64	23.45	8.25	56.99
128	22.75	4.32	36.08

a Execution configuration: 2 MPI processes per node and 3 GPU per MPI process. The number of nodes refers only to Summit nodes running worker processes.

**Table 10:** Strong Scaling Benchmark: Times in Seconds of Three Representative Tensor Contractions with 840 AO, 840 Virtual, and 280 Occupied Spinors^a^

no. of nodes	contraction 1	contraction 2	contraction 3
128	87.66	18.11	177.13
256	71.88	10.32	125.52
512	80.64	7.70	89.11

a Execution configuration: 2 MPI processes per node and 3 GPU per MPI process. The number of nodes refers only to Summit nodes running worker processes.

### 5.4 EFG of Uranium in the Uranyl Tris-nitrate Complex

**Figure 4:**
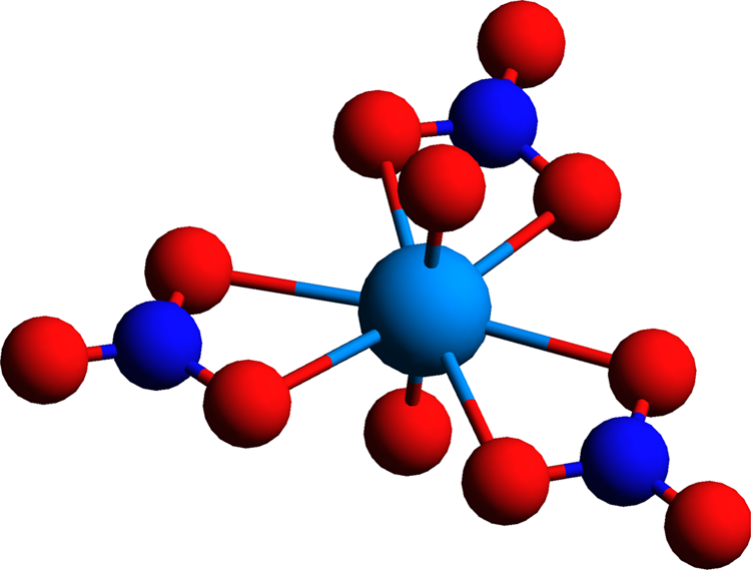
Structure of the uranyl complex derived from the X-ray structure for the RbUO_2_(NO_3_)_3_ crystyal.^67^

As a final example of possible applications, we now turn our attention to the calculation of the *q*_*zz*_ component of the EFG tensor on the uranium atom for the [UO_2_(NO_3_)_3_]^−^ complex, for which there are experimental values^97^ in the solid state. Initial theoretical investigations of EFGs for actinyl species focused on the bare uranyl ion (UO_2_^2+^),^19,20^ where it was found that a qualitative agreement with the experiment was only achieved if the effect of the equatorial ligands was taken into account (even if through point-charge embedding^19^). These studies nevertheless revealed that the U *q*_*zz*_ value had a dominant contribution from the so-called U(6p) core-hole, arising from the depletion of charge because of the overlap between the O(2p) and the high-lying antibonding U(6p_σ_) + O(2s) spinors (Figure 4).

Explicit inclusion of the contributions from the equatorial ligands to the U *q*_*zz*_ value and the associated analysis of orbital contributions to the EFG was, to the best of our knowledge, first performed by Belanzoni and co-workers,^98^ employing the BP86 generalized gradient approximation (GGA) functional, the ZORA-4 Hamiltonian, and QZ4P bases. They have, first, identified that the U(6p) core-hole yielded a positive contribution to the EFG, though in bare uranyl these were offset by negative contributions due to the nonspherical electron distribution in the valence 5f shell caused by the U–O bonding. Moreover, positive contributions due to the ligands arise from the tails of the U(6p) spinor, which extends significantly to the region of the nitrate ligands, as well as from electron donation by the nitrate groups into U(5f_ϕ_, 6d_δ_), which have lobes in the equatorial plane. These calculations were found to underestimate the U *q*_*zz*_ experimental value by around 4 au.

More recently, Autschbach and co-workers^99^ have employed the X2C-1e Hamiltonian, triple zeta quality basis sets (U, ANO(-h); light atoms, TZVPP), and density functional approximations (DFAs) including Hartree–Fock exchange, such as B3LYP and CAMB3LYP, to revisit the U *q*_*zz*_ on the [UO_2_(HCO_3_)_3_]^−^ complex that resembles fairly well the structural motif in [UO_2_(NO_3_)_3_]^−^, though for which, unfortunately, we are not aware of any experimental values. These results have demonstrated the importance of accounting for picture change effects in the representation of the EFG operator (increases in U *q*_*zz*_ of around 8 au, fairly consistent among the different methods), as well as the importance of including Hartree–Fock exchange in the DFAs from going from GGAs^98^ to hybrids^99^ for obtaining larger values for U *q*_*zz*_. They have also confirmed the large effect of the ligands on U *q*_*zz*_ found by Belanzoni and co-workers, as U *q*_*zz*_ goes from a negative value (between −8 and −7 au depending on the DFA) in bare uranyl to a positive one (see Table 12).

Though the results by Autschbach and co-workers suggest that DFAs would work rather well in this case, it is well known in the literature^100^ that it can be difficult for these to correctly represent EFGs for transition metals^101,102^ or lanthanides,^79^ and as such it would be highly desirable to perform EFG calculations at the coupled cluster level more routinely, if not to provide benchmark values for systems larger than diatomics or triatomics. In this respect, our calculations are, to the best of our knowledge, the first effort to obtain EFGs at the CCSD level for uranyl species while explicitly including the equatorial ligands—in the pioneering calculation by de Jong and co-workers,^19^ the structure of uranyl was investigated with CC, but the EFG was only calculated at the Hartree–Fock level. As such, our calculations are also the first to investigate the effects of increasing the number of correlated occupied and virtual spinors on the EFG values of uranyl complexes.

Our results are shown in Tables 11 and 12. We note that we have restricted ourselves to the X2C-AMFI Hamiltonian to remain close to the setup of prior calculations.^99^ Furthermore, due to constraints on the available memory and other resources, we were only able to carry out calculations with significantly extended virtual spaces for double zeta calculations. From Table 11, the first interesting result is the behavior of the T_1_ diagnostic. For the smallest calculation (1), we have a value slightly greater than 0.012, which is higher than what is usually observed for molecules containing light elements but in line with our experience^7,61,68,103^ of CCSD calculations on heavy elements in which we did not correlate electrons below the U 5d shell. When we increase the number of correlated electrons, the T_1_ value progressively decreases and reaches around 0.009 for the calculations in which all electrons are correlated (4–7). This would suggest, therefore, that in prior calculations the relatively high T_1_ values (with respect to the prescriptions originally put forth for light-element molecules) arise mostly due to an incomplete occupied correlating space, instead of being the sign of a growing multireference character for the heavy elements.

**Table 11:** Comparison of Times to Solution (TTS, in Hours, Equal to the Total Wall Time for Each Calculation and the Part Spent in the T and Λ Equations), T_1_ Diagnostic, and *q*_*zz*_ Component of the EFG Tensor (in Atomic Units) for CCSD Expectation Value Calculations for the Uranyl Tris-nitrate Complex ([UO_2_(NO_3_)_3_]^−^) with the X2C-AMFI Hamiltonian^a^

								TTS			
**calculation**	**O**	***x*_o_**	**V**	***x*_v_**	**M**	**C**	**NormC**	**total**	**T + Λ**	**T1**	***q*_*zz*_**
dyall.v2z											
1	106	0.53	534	0.42	160	9	1.00	3h34	2h55	0.0126	10.02
2	156	0.77	534	0.42	160	20	2.17	5h33	5h06	0.0103	9.70
3	156	0.77	818	0.64	360	109	5.30	11h21	10h43	0.0102	8.48
4	202	1.00	534	0.42	400	33	1.45	8h02	7h29	0.0091	9.73
5	202	1.00	680	0.53	2050	87	0.75	12h28	11h58	0.0090	8.89
6	202	1.00	818	0.64	2050	183	1.56	18h23	17h49	0.0089	8.59
7	202	1.00	896	0.68	2050	263	2.25	23h04	22h21	0.0089	8.54
dyall.v3z											
8	106	0.53	694	0.33	480	26	0.95	6h	4h08	0.0139	10.29
9	156	0.72	694	0.33	480	57	2.06	10h24	8h02	0.0114	10.04
10	156	0.72	944	0.46	2050	193	1.65	17h48	15h44	0.0104	9.99

a O, number of occupied spinors correlated; V, number of virtual spinors correlated; M, number of ranks in the parallel calculation; C, cost estimate for the calculation (scaled by 1.0 × 10^14^); NormC, cost divided by the number of ranks and normalized with respect to the value for the smallest calculation. See the text for details.

Concerning the value of *q*_*zz*_, we have fairly large variations going from the calculations in which the correlation space is rather small (1) to the largest calculation performed (7). Comparing calculations in which the number of occupied spinors is increased but the number of virtual spinors is kept constant (1 and 2 and 1 and 4) to those in which the reverse is true (2 and 3), we have that the size of the virtual space is the most significant variable to control. From our calculations, we observe that it is only after including roughly 60% of the virtual space in the correlating space (more than 680 virtual spinors for double zeta bases) that the *q*_*zz*_ values start to converge to a value around 8.5 au, and though we have a much more limited set of data for triple zeta bases, we seem to observe a similar pattern, with rather large variations in the *q*_*zz*_ values with a change in the number of correlated virtual spinors. Unfortunately, with the largest calculations we carried out, we are not able to include more than roughly 45% of the virtual space in the correlating space, which appears not to be sufficient for obtaining a converged *q*_*zz*_ value.

Strictly speaking, the calculations presented in Table 11 cannot be used to characterize the strong scaling of the code. This is because the memory required to store the two-electron integral tensor grows sharply as we increase the number of correlated occupied and virtuals, requiring that the number of Summit nodes used was changed for each run to fit this tensor in. It is nevertheless possible to extract some information on the code’s weak scaling. To do so, we define the following two metrics: (a) the cost of calculation *n*, taken to be proportional to the number of operations of the costlier contractions for both amplitude and Λ equations (*C*[*n*] = *O*^2^*V*^4^[*n*], with *O* being the number of correlated occupied spinors and *V* being the number of correlated virtual spinors) and (b) cost per rank *M* employed in the parallel calculation, normalized by the cost of calculation 1, the smallest considered here (NormC[*n*] = *C*[*n*]/(M*C[1])). We see that the significant cost increase resulting from the augmentation of the occupied and virtual spaces (a 28-fold increase from calculation 1 to calculation 7) can be offset by an increase in the number of ranks in the parallel calculation (a 13-fold increase comparing the same calculations) such that the time to the solution remains within reasonable bounds (less than 24 h). At the same time, NormC for the different calculations remains, with a few exceptions, between 1 and 2, and that including both the double and triple zeta calculations. This indicates that the code can handle high workloads in roughly the same manner as it does for much smaller workloads.

Finally, in Table 12, we compare our best CCSD results to uncorrelated (Hartree–Fock), DFT calculations performed with DIRAC for hybrid DFAs, to the results from Belanzoni and co-workers^98^ and Autschbach and co-workers,^99^ and to the experiment.^97^ We observe that the Hartree–Fock calculations provide largely overestimated values with respect to the experiment, with differences of above 6 au for the double, triple, and quadruple zeta bases, whereas all DFT calculations provide somewhat underestimated values—among the functionals compared, B3LYP fares the worst (differences from −1.74 to −1.49 au), while PBE0 (differences from −0.80 and −0.53 au) fares slightly better than CAMB3LYP (differences from −1.14 and −0.88 au). Taken together, these results indicate that for the mean-field approaches, an increase in basis set quality translates into an increase in *q*_*zz*_ of slightly under 0.2 au going from double to triple zeta, with an additional increase of 0.1 au when going from triple to quadruple zeta and that all DFT results appear to move in the direction of the experiment. We observe that our Hartree–Fock and DFT values are slightly smaller than those obtained by Autschbach and co-workers,^99^ though a somewhat larger discrepancy is seen for CAMB3LYP than that for B3LYP or Hartree–Fock, which suggests something other than purely the difference in the molecular system could be at play here and that the very good agreement to experiment with CAMB3LYP in the literature seems fortuitous.

**Table 12:** Comparison between Electronic Structure Methods and Experimental Results for the *q*_*zz*_ Component of the EFG Tensor for the Uranyl Tris-nitrate Complex ([UO_2_(NO_3_)_3_]^−^) for Basis Sets of Different Cardinal Numbers^b^

method	2z	3z	4z
HF	14.67	14.85	14.97
B3LYP	6.64	6.79	6.89
CAMB3LYP	7.24	7.40	7.50
PBE0	7.58	7.75	7.85
CCSD	8.54		
ref (98), BP86, ZORA-4			4.11
ref (99)^a^, HF, X2C-1e		15.17	
ref (99)^a^, B3LYP, X2C-1e		6.81	
ref (99)^a^, CAMB3LYP, X2C-1e		8.33	
exp^97^	8.38 ± 0.13		

a The coupled cluster calculation employs 202 occupied and 896 virtual spinors. All values in are atomic units. The X2C-AMFI Hamiltonian is employed in all calculations.

b Calculations on [UO_2_(HCO_3_)_3_]^−^.

Our best double zeta CCSD result (calculation 7) shows only a slight overestimation (0.16 au) with respect to the experiment, which is a significantly better result than any of the mean-field ones. That said, since we were not able to perform accurate calculations with more flexible basis sets and, from the DFT trends, it would not be surprising that these results, compared to future calculations, would be found to underestimate the *q*_*zz*_ value at least a few tenths of an atomic unit, pointing to an overall overestimation of the experimental results at the CCSD level. Once we implement approaches that allow us to efficiently truncate our (artificially, due to the use of uncontracted basis sets) large virtual spaces without the loss of accuracy, we intend to revisit this question.

## 6 Conclusions

A reimplementation of the Kramers-unrestricted coupled cluster method was presented and shown to be able to exploit hundreds of GPU-accelerated nodes while correctly reproducing the results of the prior implementation for a range of test cases. With this implementation, which currently does not exploit any rank reduction techniques or other approximations, we were already able to investigate systems for which about 200 electrons and around 1000 virtual molecular spinors were taken into account in the correlated calculations.

Current functionalities include CC2, CCD, and CCSD wave fuctions and perturbative triples corrections to the ground-state. Ground-state expectation values are also available for CCD and CCSD through the computation of one-body density matrices. The code is now interfaced to the DIRAC and ReSpect packages, but interfaces to other software packages employing Gaussian-type atomic orbitals can be implemented in a straightforward manner as they only require functionality to read in the molecular spinors.

Unlike the original implementation, this reimplementation does not exploit double point group symmetry. For the aforementioned functionality, this is a disadvantage for small, highly symmetric systems (i.e., around 10 atoms or 100 electrons), though we consider that in practice this shortcoming is offset by large-scale parallelization that allows us to treat such systems upon distortions (bent/twisted configurations) with triple or quadruple zeta basis sets without hitting the wall of transitioning from real to complex algebra (as is the case in the original code when symmetry is reduced), inefficient communication over hundreds of nodes, and the extensive use of disk storage of intermediate quantities. For larger systems, which often have little to no symmetry, the current implementation makes correlated calculations feasible.

We have employed the code to investigate the properties of different heavy-element complexes: ionization energies for lanthanide monofluorides (LaF, EuF, and YbF), energies of formation of gold–argon clusters of different sizes, and the electric field gradient (*q*_*zz*_^*U*^) at the uranium nucleus in a uranyl tris-nitrate complex.

For the lanthanide monofluorides, we have shown that we need to correlate at least the Ln (4s4p4d4f) electron shell to obtain converged ionization energies. We observed that for LaF and EuF, the perturbative triples-corrected ionization energies for the triple zeta quality basis sets are already within the experimental error bounds, with adiabatic values showing a slightly better agreement to the experiment than vertical ones. For YbF, we have encountered the same issue previously described in the literature, in that spin–orbit-coupled calculations show a surprisingly large *T*_1_ diagnostic value around the equilibrium distance, which makes perturbative triples results unreliable, but we see a smooth convergence of the CCSD values toward the experimental values.

For the gold–argon clusters, substantial binding energy is obtained at the coupled cluster level for closed-shell noble metal Au^+^ and noble gas Ar, with negligible values at the Hartree–Fock level (below 0.6 eV) and overestimation of the binding energy up to 0.5 eV for MP2. Therefore, a selection of a reliable method is important. For AuAr_4_^+^, two different structures that are close in energy have been computed and the 3D structure is about 0.2/0.3 eV lower than the 2D structure at the CCSD/CCSD(T) level.

For the uranyl tris-nitrate complex, we have found that CCSD wave functions are capable of providing *q*_*zz*_^U^ values in very good agreement to the experiment, with non-negligible improvements over DFT calculations. This improvement, however, appears to come at the cost of including high-lying virtual spinors in the CCSD calculation. We have been limited to performing CCSD calculations with double zeta basis due to the number of virtual spinors that appears to be required to converge the *q*_*zz*_^U^ value. While the DFT results suggest non-negligible basis set effects due to the use of uncontracted basis sets, for reasons of computational cost, it was not possible to include enough high-lying virtual spinors in triple zeta calculations and therefore the calculations we were able to perform are still far from converged. We are currently working on implementing approaches to compress the virtual space while retaining precision in the correlated treatment to enable efficient calculations with larger basis sets. We expect that these will allow us not only to revisit this system in the near future but also enable calculations on significantly larger systems.

## Supporting Information Available

Relevant equations for the implementation, more detailed comparison between the current implementation and RELCCSD results, tables determining the active space of LaF and EuF, CCSD bond distances for AuAr^+^, DFT results for (UF_6_)_2_, and details on setting up the input and code examples for the usage of TAL-SH and ExaTENSOR (PDF)
