# Supplementary material for: Implementation of Relativistic Coupled Cluster Theory for Massively Parallel GPU-Accelerated Computing Architectures
Source: J Chem Theory Comput. 2021 Aug 9;17(9):5509–29. doi: 10.1021/acs.jctc.1c00260 (PMC8444343; doi:10.1021/acs.jctc.1c00260)
Supplement: Supplementary file 1 — ct1c00260_si_001.pdf [file ct1c00260_si_001.pdf]

Supporting Information - Implementation of relativistic coupled cluster theory for massively parallel GPU-accelerated computing architectures

Johann V. Pototschnig,[*,†] Anastasios Papadopoulos,[*,†,⊥] Dmitry I. Lyakh,[‡] Michal Repisky,[¶] Loïc. Halbert,[§] André Severo Pereira Gomes,[*,§] Hans Jørgen Aa. Jensen,[*,‖] and Lucas Visscher[*,†]

†*Department of Chemistry and Pharmaceutical Science, Faculty of Science, Vrije Universiteit Amsterdam, de Boelelaan 1083, 1081 HV Amsterdam, The Netherlands*

‡*National Center for Computational Sciences, Oak Ridge National Laboratory, Oak Ridge TN, USA*

¶*Hylleraas Centre for Quantum Molecular Sciences, Department of Chemistry, UiT The Arctic University of Norway, N-9037 Tromsø, Norway*

§*Université de Lille, CNRS, UMR 8523 – PhLAM – Physique des Lasers, Atomes et Molécules, F-59000 Lille, France*

‖*Department of Physics, Chemistry and Pharmacy, University of Southern Denmark, DK-5230 Odense M, Denmark*

⊥*Department of Molecular Theory and Spectroscopy, Max-Planck-Institut für Kohlenforschung, Kaiser-Wilhelm-Platz 1, 45470 Mülheim an der Ruhr, Germany*

E-mail: j.v.pototschnig@vu.nl; papadopoulos@kofo.mpg.de; andre.gomes@univ-lille.fr; hjj@sdu.dk; l.visscher@vu.nl

This is the supporting information for the manuscript entitled: "Implementation of relativistic coupled cluster theory for massively parallel GPU-accelerated computing architectures" by J. V. Pototschnig, A. Papadopoulos, D. I. Lyakh, M. Repisky, L.Halbert, A. S. P. Gomes, H. J. Aa. Jensen, and L. Visscher.

At first, section 1 contains the working equations implemented in the new code. In section 2 we compared the results for small test systems with the RELCCSD reference implementation in DIRAC.[1] Subsequently, tables to determine the active occupied and virtual spinors for LaF and EuF are shown in section 3. Next, there are some results for the structure of molecules, first for for $AuAr^+$ (section 4), then for $(UF_6)_2$ (section 5). Section 6 contains a list of the input options in the new code and an example for an input file. In the following two parts some code examples are given for ExaTENSOR (section 7) and TAL-SH (section 8) and their usage is discussed. Finally, we discuss the different integral transformation routines available in the ExaTensor part of the code and present an argument for the default in section 9.

1 Working equations

1.1 CCSD amplitude equations

The working equations for coupled cluster presented in ref. 2 were optimized to reduce the operation count. In the current implementation all the tensors are kept in memory and, therefore, it is desirable to avoid large intermediates, even if the number of operations is slightly increased. The B intermediate in ref. 2 has a size of n_{vir}^4 and can be avoided by

using the following equations:

$$P_{pq}^{-} f(p,q) = f(p,q) - f(q,p) \tag{1}$$

$$\tau_{ij}^{ab} = t_{ij}^{ab} + P_{ij}^{-}\left(t_i^a t_j^b\right) \tag{2}$$

$$H_c^a = F_c^a + \frac{1}{2}\sum_{kld} V_{cd}^{kl}\tau_{kl}^{da} \tag{3}$$

$$G_c^a = H_c^a + \sum_{kd} V_{cd}^{ak} t_k^d - \sum_k F_c^k t_k^a \tag{4}$$

$$H_i^k = -F_i^k + \frac{1}{2}\sum_{lcd} V_{cd}^{kl}\tau_{li}^{cd} \tag{5}$$

$$G_i^k = H_i^k - \sum_{lc} V_{ic}^{kl} t_l^c - \sum_c F_c^k t_i^c \tag{6}$$

$$H_c^k = F_c^k + \sum_{ld} V_{cd}^{kl} t_l^d \tag{7}$$

$$H_{ci}^{ak} = V_{ci}^{ak} + \sum_d V_{cd}^{ak} t_i^d - \sum_l V_{ic}^{kl} t_l^a + \sum_{ld} V_{cd}^{kl}\left(\frac{1}{2}t_{li}^{ad} + t_l^a t_i^d\right) \tag{8}$$

$$C_{ij}^{kb} = V_{kb}^{ij\dagger} + \sum_c P_{ij}^{-}\left(V_{ci}^{bk} t_j^c\right) - \frac{1}{2}\sum_{cd} V_{cd}^{bk}\tau_{ij}^{cd} \tag{9}$$

$$A_{ij}^{kl} = V_{ij}^{kl} + \sum_c P_{ij}^{-}\left(V_{ic}^{kl} t_j^c\right) + \frac{1}{2}\sum_{cd} V_{cd}^{kl}\tau_{ij}^{cd} \tag{10}$$

$$\begin{aligned} S_{ij}^{ab} &= \sum_{kc} P_{ij}^{-} P_{ab}^{-}\left(H_{ci}^{ak} t_{jk}^{cb}\right) \\ &+ \sum_k P_{ji}^{-} G_j^k t_{ik}^{ab} + \sum_c P_{ij}^{-}\left(V_{ab}^{cj\dagger} t_i^c\right) + \sum_c P_{ab}^{-} G_c^a t_{ij}^{cb} + \sum_k P_{ab}^{-}\left(C_{ij}^{ka} t_k^b\right) \\ &+ \frac{1}{2}\sum_{kl} A_{ij}^{kl}\tau_{kl}^{ab} + \frac{1}{2}\sum_{cd} V_{cd}^{ab}\tau_{ij}^{cd} + V_{ab}^{ij\dagger} \end{aligned} \tag{11}$$

$$\begin{aligned} S_i^a &= F_a^{i\dagger} + \sum_c H_c^a t_i^c + \sum_k H_i^k t_k^a + \sum_{kc} H_c^k t_{ik}^{ac} \\ &+ \sum_k\left(\sum_c\left(H_c^k - 2F_c^k\right)t_i^c\right)t_k^a + \frac{1}{2}\sum_{kcd} V_{cd}^{ak}\tau_{ik}^{cd} + \frac{1}{2}\sum_{klc} V_{ic}^{kl}\tau_{kl}^{ca} - \sum_{kc} V_{ci}^{ak} t_k^c \end{aligned} \tag{12}$$

1.2 CC2 amplitude equations

In the CC2 method[3] the T_1 amplitude equations in 1.1 are unchanged whereas the T_2 amplitude equations are given by

$$\begin{aligned} S_{ij}^{ab} =& V_{ij}^{ab} + \sum_e P_{ij}^- t_i^e W_{ej}^{ab}{}_{CC2} - \sum_m P_{ab}^- t_m^a W_{ij}^{mb}{}_{CC2} \\ &+ \sum_{m<n} P_{ab}^- t_m^a t_n^b W_{ij}^{mn}{}_{CC2} + \sum_{e<f} P_{ij}^- t_i^e t_j^f W_{ef}^{ab}{}_{CC2} \\ &+ \sum_e P_{ab}^- f_e^b t_{ij}^{ae} - \sum_m P_{ij}^- f_j^m t_{im}^{ab} \end{aligned} \tag{13}$$

with :

$$W_{ij}^{mn}{}_{CC2} = V_{ij}^{mn} + \sum_e P_{ij}^- t_j^e V_{ie}^{mn} + \frac{1}{4} \sum_{ef} P_{ij}^- t_i^e t_j^f V_{ef}^{mn} \tag{14}$$

$$W_{ef}^{ab}{}_{CC2} = V_{ef}^{ab} - \sum_m P_{ab}^- t_m^b V_{ef}^{am} + \frac{1}{4} \sum_{mn} P_{ab}^- t_m^a t_n^b V_{ef}^{mn} \tag{15}$$

$$W_{ej}^{ab}{}_{CC2} = V_{ej}^{ab} - \frac{1}{2} \sum_m P_{ab}^- t_m^a V_{ej}^{mb} \tag{16}$$

$$W_{ij}^{mb}{}_{CC2} = V_{ij}^{mb} + \frac{1}{2} \sum_e P_{ij}^- t_i^e V_{ej}^{mb} \tag{17}$$

1.3 Perturbative triples

In contrast to ref. 2 the full term is used, not only the upper triangle. The following expression needs to be evaluated for the older fourth order term

$$E^{(+T)} = -\sum_{i,j,k}^{n_{occ}} \sum_{a,b,c}^{n_{vir}} \frac{\left(W_{ijk}^{abc} + W_{ijk}^{bca} + W_{ijk}^{cab}\right)\left(W_{ijk}^{abc} + W_{ijk}^{bca} + W_{ijk}^{cab}\right)}{36\, D_{ijk}^{abc}}, \tag{18}$$

where D_{ijk}^{abc} is the energy denominator computed from the spinor energies and the intermediate is defined in following manner

$$W_{ijk}^{abc} = \sum_e \langle ie||ab\rangle \, t_{jk}^{ec} + \sum_e \langle je||ab\rangle \, t_{ki}^{ec} + \sum_e \langle ke||ab\rangle \, t_{ij}^{ec} + \sum_m \langle ij||am\rangle \, t_{km}^{bc} + \sum_m \langle jk||am\rangle \, t_{im}^{bc} + \sum_m \langle ki||am\rangle \, t_{jm}^{bc}. \tag{19}$$

The fifth order correction is given by

$$E^{((T))} = E^{(+T)} + \sum_{i,j,k}^{n_{occ}} \sum_{a,b,c}^{n_{vir}} \frac{\left(W_{ijk}^{abc} + W_{ijk}^{bca} + W_{ijk}^{cab}\right)\left(Z_{ijk}^{abc} + Z_{ijk}^{cab} + Z_{ijk}^{bca}\right)}{36\, D_{ijk}^{abc}}, \tag{20}$$

using the intermediate

$$Z_{ijk}^{abc} = \langle ij||ab\rangle^* \, t_k^c + t_{ij}^{ab\,*} f_k^c + \langle jk||ab\rangle^* \, t_i^c + t_{jk}^{ab\,*} f_i^c + \langle ki||ab\rangle^* \, t_j^c + t_{ki}^{ab\,*} f_j^c. \tag{21}$$

An alternative fifth order expression[4] is defined by

$$E^{(-T)} = E^{(+T)} + \sum_{i,j,k}^{n_{occ}} \sum_{a,b,c}^{n_{vir}} \frac{\left(W_{ijk}^{abc} + W_{ijk}^{bca} + W_{ijk}^{cab}\right)\left(Y_{ijk}^{abc} + Y_{ijk}^{cab} + Y_{ijk}^{bca}\right)}{36}, \tag{22}$$

applying the expression

$$Y_{ijk}^{abc} = \frac{1}{3}\left(t_i^a t_j^b t_k^c - t_j^a t_i^b t_k^c + t_j^a t_k^b t_i^c - t_k^a t_j^b t_i^c + t_k^a t_i^b t_j^c - t_i^a t_k^b t_j^c\right) + t_{ij}^{ab} t_k^c + t_{jk}^{ab} t_i^c + t_{ki}^{ab} t_j^c. \tag{23}$$

All three expressions are evaluated in our implementation so that a user of the program may easily assess the resulting differences in energies.

1.4 CCSD lambda equations

This equations are close to the equations presented in ref. 5, there was a slight error in the equations in the reference, which is corrected here. These are the corrected equations:

$$W^{ij}_{mn} = V^{ij}_{mn} + P^{-}_{mn}\sum_{e} V^{ij}_{en} t^{e}_{m} + \sum_{e<f} V^{ij}_{ef}\tau^{ef}_{mn} \tag{24}$$

$$W^{mb}_{ej} = V^{mb}_{ej} + \sum_{f} V^{mb}_{ef} t^{f}_{j} - \sum_{n} V^{mn}_{ej} t^{b}_{n} \tag{25}$$

$$- \sum_{nf} V^{mn}_{ef}(t^{fb}_{jn} + t^{f}_{j} t^{b}_{n}) \tag{26}$$

$$W^{ef}_{am} = V^{ef}_{am} + P^{-}_{ef}\sum_{ng} V^{en}_{ag} t^{fg}_{mn} + \sum_{g} W^{ef}_{ag} t^{g}_{m} \tag{27}$$

$$+ \sum_{n} \overline{F}^{n}_{a} t^{ef}_{mn} + \sum_{n>o} V^{no}_{am}\tau^{ef}_{no} - P^{-}_{ef}\sum_{n} \overline{W}^{nf}_{am} t^{e}_{n} \tag{28}$$

$$\mathcal{G}^{e}_{a} = - \sum_{f,m<n} \lambda^{mn}_{af} t^{ef}_{mn} \tag{29}$$

$$\mathcal{G}^{i}_{m} = \sum_{n,e<f} \lambda^{in}_{ef} t^{ef}_{mn} \tag{30}$$

$$0 = V^{ij}_{ab} + P^{-}_{ab}\sum_{e} \lambda^{ij}_{ae}\overline{F}^{e}_{b} - P^{-}_{ij}\sum_{m} \lambda^{im}_{ab}\overline{F}^{j}_{m} + \sum_{m>n} \lambda^{mn}_{ab} W^{ij}_{mn} + P^{-}_{ij} P_{ab}\sum_{me} \lambda^{im}_{ae} W^{je}_{bm} \tag{31}$$

$$+ P^{-}_{ab}\sum_{e} V^{ij}_{ae}\mathcal{G}^{e}_{b} - P^{-}_{ab}\sum_{m} \lambda^{m}_{a} W^{ij}_{mb} - P^{-}_{ij}\sum_{m} V^{im}_{ab}\mathcal{G}^{j}_{m} + P^{-}_{ij}\sum_{e} \lambda^{i}_{e} W^{ej}_{ab} \tag{32}$$

$$+ P^{-}_{ij} P^{-}_{ab}\lambda^{i}_{a}\overline{F}^{j}_{b} + \sum_{e>f} \lambda^{ij}_{ef} W^{ef}_{ab} \tag{33}$$

$$0 = \overline{F}^{a}_{i} + \sum_{e} \lambda^{i}_{e}\overline{F}^{e}_{a} - \sum_{m} \lambda^{m}_{a}\overline{F}^{i}_{m} - \sum_{mn} \mathcal{G}^{n}_{m} W^{mi}_{na} - \sum_{ef} \mathcal{G}^{f}_{e} W^{ei}_{fa} \tag{34}$$

$$+ \sum_{me} \lambda^{m}_{e} W^{ie}_{am} - \sum_{m>n,e} \lambda^{mn}_{ae} W^{ie}_{mn} + \sum_{m,e<f} \lambda^{im}_{ef} W^{ef}_{am} \tag{35}$$

$$\overline{F}^{e}_{a} = f^{e}_{a} - \sum_{m} f^{m}_{a} t^{e}_{m} + \sum_{mf} V^{me}_{fa} t^{f}_{m} - \sum_{m>n,f} V^{mn}_{af}\tau^{ef}_{mn} \tag{36}$$

The full list including intermediates can be found in ref. 5

1.5 Lagrange 1-body density matrix

$$\gamma_{ij} = \frac{1}{2}\sum_{mef} t_{im}^{ef}\lambda_{ef}^{mj} - \sum_{e} t_i^e \lambda_e^j \tag{37}$$

$$\gamma_{ia} = \lambda_a^i \tag{38}$$

$$\gamma_{ai} = t_i^a + \sum_{me}\lambda_e^m(t_{im}^{ae} - t_i^e t_m^a) - \frac{1}{2}\sum_{mnef}\lambda_{ef}^{mn}(t_{in}^{ef}t_m^a + t_i^e t_{mn}^{af}) \tag{39}$$

$$\gamma_{ab} = \frac{1}{2}\sum_{mne} t_{mn}^{ae}\lambda_{be}^{mn} + \sum_{m} t_m^a \lambda_b^m \tag{40}$$

The density matrix is symmetrized before use in property calculations.

2 Verify accuracy by comparing to RELCCSD

2.1 Energy

At first we want to check that the correct numbers are produced by the code. H_2O and LiO were selected as small examples for an closed- and open-shell molecule, respectively. The differences between the new TAL-SH implementation and the RELCCSD results are collected in table 1. Correspondingly, table 2 contains the results for ExaTENSOR. If one looks at the tables the differences between the closed- and open-shell system are noticeable. For the closed-shell molecule the errors are below the convergence threshold of 1.0E-8, but

Table 1: Differences between RELCCSD and TAL-SH for H_2O and LiO obtained for an convergence threshold of 1.0E-8. Values are in Hartree.

Molecule	Δ CCSD	Δ CCSD+T	Δ CCSD(T)	Δ CCSD-T
H2O(DZ)	5.10E-11	4.80E-11	4.50E-11	4.50E-11
H2O(TZ)	1.10E-11	2.00E-11	2.10E-11	2.00E-11
H2O(QZ)	1.20E-11	2.80E-11	2.60E-11	2.50E-11
LiO(DZ)	1.00E-08	1.30E-07	2.20E-08	1.30E-07
LiO(TZ)	1.00E-08	8.20E-07	2.20E-07	8.20E-07
LiO(QZ)	9.90E-09	6.10E-07	1.40E-07	6.10E-07

Table 2: Differences between RELCCSD and ExaCorr for H_2O and LiO obtained for an convergence threshold of 1.0E-8. Values are in Hartree.

Molecule	Δ CCSD	Δ CCSD+T	Δ CCSD(T)	Δ CCSD-T
H2O(DZ)	5.10E-11	4.80E-11	4.50E-11	4.50E-11
H2O(TZ)	1.10E-11	2.10E-11	2.10E-11	2.00E-11
H2O(QZ)	2.20E-11	3.80E-11	3.60E-11	3.50E-11
LiO(DZ)	1.00E-08	2.00E-07	1.80E-07	2.00E-07
LiO(TZ)	1.00E-08	6.00E-07	1.00E-06	6.00E-07
LiO(QZ)	9.90E-09	4.40E-07	8.60E-07	4.40E-07

for LiO this only holds for the CCSD energy. The perturbative triples show slightly larger deviations.

In order to test a larger system with different symmetries we selected $CuAr_n^+$ which was recently studied.[6] The agreement is satisfactory, see table 3. In the recent publication also the

Table 3: Differences between RELCCSD and ExaCorr for $CuAr_n$ obtained for an convergence threshold of 1.0E-7. Values are in Hartree.

Molecule	Δ CCSD	Δ CCSD+T	Δ CCSD(T)	Δ CCSD-T
$CuAr_1^+$	1.5E-07	1.4E-07	1.5E-07	1.5E-07
$CuAr_2^+$	1.8E-07	1.8E-07	1.8E-07	1.8E-07
$CuAr_3^+$	2.0E-07	2.0E-07	2.0E-07	2.0E-07

open-shell molecules $Cu_2Ar_n^+$ were studied besides $CuAr_n^+$. In this case a direct comparison is not possible since in RELCCSD symmetry was used to deal with spinor degeneracy obtained the SCF level. In ExaCorr the spinor energies are recomputed, but this is not sufficient and non-convergence is observed in the CC iterations. A solution is to use a level shift for the virtual spinors, which results in convergence. Nevertheless, there is a difference in the binding energy of the two approaches of about 1.0E-3 Hartree.

2.2 Properties

The properties were checked by comparing the two implementations (TAL-SH, ExaTENSOR) to the well tested DIRAC module. A non-symmetric molecule was selected and the results are listed in table 4. As can be see satisfactory agreement was obtained. In order to

Table 4: Differences between RELCCSD, ExaTENSOR and TAL-SH properties for CHFClBr obtained for an convergence threshold of 1.0E-8. Dipole moments and electric field gradients are in atomic units, the nuclear quadrupole coupling constant in MHz.

Property	xyz	RELCCSD	TAL-SH	ExaTENSOR	Δ (TAL-SH)	Δ (ExaTENSOR)
DM	X	-0.32275	-0.32275	-0.32275	< 1E-10	< 1E-10
	y	-0.37547	-0.37547	-0.37547	< 1E-10	< 1E-10
	z	-0.08469	-0.08469	-0.08469	< 1E-10	< 1E-10
EFG Br	qxx	-4.30878	-4.30878	-4.30878	1E-09	1E-09
	qyy	-4.07288	-4.07288	-4.07288	1E-09	1E-09
	qzz	8.38165	8.38165	8.38165	-2E-09	-2E-09
EFG Cl	qxx	4.04825	4.04825	4.04825	-2E-09	-2E-09
	qyy	-1.95116	-1.95116	-1.95116	1E-09	1E-09
	qzz	-2.09708	-2.09708	-2.09708	1E-09	1E-09
EFG F	qxx	-1.60295	-1.60295	-1.60295	< 1E-10	< 1E-10
	qyy	3.06056	3.06056	3.06056	< 1E-10	< 1E-10
	qzz	-1.45760	-1.45760	-1.45760	< 1E-10	< 1E-10
EFG C	qxx	-0.39569	-0.39569	-0.39569	-2E-10	-2E-10
	qyy	0.27723	0.27723	0.27723	< 1E-10	< 1E-10
	qzz	0.11845	0.11845	0.11845	2E-10	2E-10
EFG H	qxx	0.30145	0.30145	0.30145	< 1E-10	< 1E-10
	qyy	-0.14711	-0.14711	-0.14711	1E-10	1E-10
	qzz	-0.15434	-0.15434	-0.15434	< 1E-10	< 1E-10
NQCC Br	Xxx	-316.88	-316.88	-316.88	9E-08	1E-07
	Xyy	-299.54	-299.54	-299.54	2E-08	4E-08
	Xzz	616.42	616.42	616.42	-1E-07	-1E-07
	Eta	0.02814	0.02814	0.02814	< 1E-10	< 1E-10
NQCC Br	Xxx	-264.75	-264.75	-264.75	7E-08	9E-08
	Xyy	-250.25	-250.25	-250.25	1E-08	3E-08
	Xzz	515.00	515.00	515.00	-9E-08	-1E-07
	Eta	0.02814	0.02814	0.02814	< 1E-10	< 1E-10
NQCC Cl	Xxx	-77.67	-77.67	-77.67	4E-08	4E-08
	Xyy	37.43	37.43	37.43	-2E-08	-2E-08
	Xzz	40.23	40.23	40.23	-2E-08	-2E-08
	Eta	0.03604	0.03604	0.03604	< 1E-10	< 1E-10
NQCC Cl	Xxx	-61.21	-61.21	-61.21	3E-08	3E-08
	Xyy	29.50	29.50	29.50	-2E-08	-2E-08
	Xzz	31.71	31.71	31.71	-2E-08	-2E-08
	Eta	0.03604	0.03604	0.03604	< 1E-10	< 1E-10
NQCC H	Xxx	0.20	0.20	0.20	< 1E-10	< 1E-10
	Xyy	-0.10	-0.10	-0.10	< 1E-10	< 1E-10
	Xzz	-0.10	-0.10	-0.10	1E-10	1E-10
	Eta	0.02400	0.02400	0.02400	< 1E-10	< 1E-10

test a large and heavier molecule UF_6 was computed, see table 5.

Table 5: Differences between RELCCSD and ExaCorr properties for UF_6 obtained for an convergence threshold of 1.0E-8. Dipole moments and electric field gradients are in atomic units, the nuclear quadrupole coupling constant in MHz.

Property	xyz	RELCCSD	ExaTENSOR
DM	X	0.00000	0.00000
	y	0.00000	0.00000
	z	0.00000	0.00000
EFG U	qxx	0.00000	0.00000
	qyy	0.00000	0.00001
	qzz	0.00000	-0.00001
EFG F	qxx	-0.37729	
	qyy	-0.37729	-0.37729
	qzz	0.75459	0.75459
NQCC U	Xxx	0.00	0.00
	Xyy	0.00	0.01
	Xzz	0.00	-0.01
	Eta	0.42310	0.55373
NQCC F	Xxx	0.00	0.00
	Xyy	0.00	0.00
	Xzz	0.00	-0.01
	Eta	0.42310	0.55373

3 Additional tables for the monofluorides

Table 6: Ionization potential in eV of LaF for different numbers of correlated spinors. The number of occupied and virtual spinors refers to the neutral molecule, one spinor changes from occupied to virtual for the cation. The ionization potential of the reference determinant in all computations was 4.9346 eV. The spinor thresholds are listed in atomic units.

threshold_{low}	threshold_{high}	nocc	nvir	% occ	% vir	CCSD
-3	40	18	136	27	44	5.9097
-20	40	36	136	55	44	5.9067
-60	40	56	136	85	44	5.9067
-300	40	64	136	97	44	5.9066
-20	2	36	74	55	24	5.8782
-20	10	36	110	55	36	5.9049
-20	40	36	136	55	44	5.9067
-20	200	36	184	55	60	5.9067
exp.						6.3±0.3 [7]

Table 7: Ionization potential in eV of EuF for different numbers of correlated spinors. The number of occupied and virtual spinors refers to the neutral molecule, one spinor changes from occupied to virtual for the cation. The ionization potential of the reference determinant in all computations was 5.0399 eV. The spinor thresholds are listed in atomic units.

threshold_{low}	threshold_{high}	nocc	nvir	% occ	% vir	CCSD
-20	26	42	218	47	41	5.460
-50	26	54	218	75	41	5.462
-200	26	62	218	86	41	5.461
-270	26	66	218	92	41	5.461
-50	26	54	218	75	41	5.462
-50	40	54	234	75	44	5.462
-50	80	54	258	75	48	5.464
-50	200	54	303	75	55	5.465
-50	500	54	326	75	61	5.465
-200	200	62	303	86	55	5.464
exp.						5.9±0.3 [8]

4 Bond distances for the gold argon cation

Table 8: $AuAr^+$ bond distances for different basis set sizes and levels of theory. They were obtained by fitting a Morse potentials to the lowest points of the potential.

basis	V	HF	MP2	CCSD	CCSD+T	CCSD(T)	CCSD-T
2z	136	2.907	2.498	2.546	2.515	2.522	2.523
3z	230	2.862	2.438	2.515	2.480	2.485	2.485
4z	400	2.862	2.414	2.505	2.465	2.471	2.471

5 DFT optimization for the uranium hexafluoride dimer

Table 9: UF_6 dimer bonding energies(eV) and distances (Å) as obtained in DFT computations. The U-F distances have been fixed for the experimental structures column, in the optimized case they range from 2.028 to 2.034 Å with the mean values listed in the table. The U-U distances were optimized in all DFT computations.

	DFT (opt. struct)			DFT (exp. struct)		
sym.	U-U	U-F	dE	U-U	U-F	dE
D2d	5.142	2.030	-0.552	5.139	1.996	-0.160
D3d	5.182	2.030	-0.526	5.144	1.996	-0.136
C2h	5.298	2.030	-0.541	5.290	1.996	-0.150

The completely optimized structures in table 9 show a U-F bond distance which is about 0.034 Å larger than the experimentally determined ones,[9] with the U-U internuclear separations also being larger in the fully optimized case. The bonding energy increases by about 0.34 eV for the fully relaxed structures.

6 Input

This section contains useful information for setting up an input file. The possible keywords for ExaCorr are listed in table 10. A definition of the occupied and virtual spinors using .OCCUPIED and .VIRTUAL is necessary. The keyword .EXATENSOR is required to activate the multi-node version, the default is to use the single node TAL-SH code.

Table 10: Keywords of the ExaCorr module

keyword	example	description
.EXATENSOR		Keyword activates the multi-node implementation using ExaTENSOR, otherwise the single-node TAL-SH implementation is used
.NOTRIPLES		keyword to deactivate triples
.LAMBDA		Solve lambda equations, needs to be activated for properties
.TALSH_BUFF	50	Maximum memory used in TAL-SH / for density matrix<br>50 GB default, set to 200 GB on summit
.CCDOUBLES		only compute CCD, default is CCSD
.CC2		CC2 calculation, energies working, properties in progress
.OCCUPIED	2..6,9,23..27	obligatory keyword, as list of MOs or energy range
.VIRTUAL	energy 0.0 20.0 0.1	obligatory keyword, as list of MOs or energy range
.MOINT_SCHEME	4	1-4 available for ExaTENSOR, 42 uses Cholesky decomposition
.OCC_BETA	2..6,9,23..27	list of beta spin MOs or energy range, alpha spin defined by .OCCUPIED, only use in open-shell computations
.VIR_BETA	energy 0.0 2.0 0.1	list of beta spin MOs or energy range, alpha spin defined by .VIRTUAL, only use in open-shell computations
.EXA_BLOCKSIZE	75	Number to branch the tensors, should be < nocc
.PRINT	0	print level
.LSHIFT	0.0D-0	Level shift
.NCYCLES	30	Set the number of CC cycles
.TCONVERG	1.0D-9	Set convergence criterria (CC iterations, lambda equations)
.CHOLESKY	1.0D-9	threshold for Cholesky decomposition

Below is an example for an input file, where ExaCorr is called by DIRAC.

```
**DIRAC
.WAVE FUNCTIONS
.PROPERTIES
**PROPERTIES
.DIPOLE
.EFG
.NQCC
**HAMILTONIAN
.X2C
**INTEGRALS
*READIN
.UNCONTRACT
**WAVE FUNCTIONS
.SCF
.EXACC
*SCF
.ERGCNV
1.0E-9
.MAXITR
50
**EXACC
.EXATENSOR
.OCCUPIED
energy -6.0 -0.4 0.001
.VIRTUAL
energy -0.4 5.0 0.001
.NOTRIPLES
.LAMBDA
.MOINT_SCHEME
1
.TCONVERG
1.0E-8
.NCYCLES
80
**MOLECULE
*SYMMETRY
.NOSYM
*BASIS
.DEFAULT
dyall.v2z
*END OF
```

An additional xyz-file specifying the coordinates of the molecule is necessary. It can then be

run by:

```
PATH_TO_DIRAC/pam --mpi=#mpi --mol=molecule.xyz --inp=dirac.inp
```

7 ExaTENSOR code examples

In this part we will give hints and examples for implementing code based on the ExaTENSOR library.

7.1 Spaces

Before the library can be started the spaces and method used in the library need to be defined. In order to set up the spaces an basis needs to be define first using the subspace_basis_t data type. The basis is set up using

```
call basis%subspace_basis_ctor(basis_size,ierr)
```

where the basis functions can be assigned a color (optional argument) to differentiate them:

```
call basis%set_basis_func(i_function,BASIS_ABSTRACT,ierr,symm=color).
```

This assignment of colors assures that the indices are not split if they have the same color. The space is then registered using the basis

```
ierr=exatns_space_register('space',basis,space_id,space,branch_factor)
```

where space_id is an integer identifying the space and space is an object of the class h_space_t. The branching factor determines in how many pieces the tensor is split up during execution and was determined by

```
branch_factor = max(2, (int(get_num_segments(int(nvir,8),
int(exa_input%exa_blocksize,8)),4) - 1)/tavp_mng_depth + 1)
```

7.2 Methods

By extending the tens_method_uni_t type user-defined methods can be created, like for example a method to initialize a tensor representing a delta function

```
type, extends(tens_method_uni_t), public:: delta_t
  integer(INTL), private :: ind(4)
  contains
    procedure, public:: delta_t_init
    procedure, public:: reset=>delta_t_reset
    procedure, public:: pack=>delta_t_pack
    procedure, public:: unpack=>delta_t_unpack
    procedure, public:: apply=>delta_t_apply
end type delta_t
```

Such a type can contain private information, like in this case an integer that determines where to put the unit values in the otherwise zero tensor. For the initalizer of the MO coefficients the coefficients themselves are stored in the type. There is one method to set this data before the method is set up, in this case delta_t_init. There is also the possibility to reset private data, but than it needs to communicated with the pack and unpack procedures. If the method is called the apply function is executed on each node. In order to get the local part of the tensor the following function is used

```
tens=tensor%get_dense_adapter(ierr)
```

The variable tens is of the tens_dense_t type and contains information about the size of the local tensor as well as an pointer to it, which can be accessed as an Fortran array

```
call c_f_pointer(tens%body_ptr,tens_fortran,tens%dims(1:tens%num_dims))
```

which can be manipulated with standard Fortran operations. It is also possible to use external libraries as for example InteRest.[10]

The methods defined in such a way have to be registered before the library is started and if there are any private variables that need to be initialized it has to be done beforehand.

```
call f_delta%delta_t_init(ind_tens)
ierr=exatns_method_register('Delta_F',f_delta)
```

During runtime of the library the private variables can be updated via

```
call f_delta%reset(ind_tens)
```

7.3 Usage

The library is then started using the command

```
ierr=exatns_start()
```

where as an optional parameter the MPI communicator from the code using the ExaTENSOR library can be passed on. A tensor first needs to be created

```
ierr=exatns_tensor_create(tensor,"tensor_name",tensor_id,tensor_root,EXA_DATA_KIND_C8)
```

, where tensor is a variable of the type tens_rcrsv_t, the name of the tensor i given by tensor_name, tensor_id is a vector containing the space id's for all the dimensions, and tensor_root determines the actual length for these dimensions. Every tensor needs to be initialized either with a user-defined method (in this case ZERO is replaced by the name of the method) or a constant (ZERO is the default value of the same type as the tensor) using the command

```
ierr=exatns_tensor_init(tensor,'ZERO')
```

.

The main operation of the code is a tensor contraction

```
ierr=exatns_tensor_contract("S(a,b,i,j)+=V(a,b,c,d)*T(c,d,i,j)",s2,vvvv,t2,scalar)
```

, where the string defines the contraction, s2/vvvv/t2 are tens_rcrsv_t tensors and the scalar allows to scale the result. The application of user-defined is also important

```
ierr=exatns_tensor_transform(tensor,method)
```

. The method can either be called by its name or passed as an object, if it is updated.

After usage the memory of a tensor is released by

```
ierr=exatns_tensor_destroy(tensor)
```

There are two ways to get values back, one is to create a scalar tensor and use the function

```
ierr=exatns_tensor_get_scalar(tensor,value)
```

. An alternative is to create a TAL-SH tensor on the main node and obtain a part of the tensor or the whole tensor by

```
ierr=exatns_tensor_get_slice(tensor,TALSH_tensor)
```

.

8 TAL-SH code examples

This part contains some hints regarding the implementations with TAL-SH. This library doesn't require a set up of spaces or methods before it starts, as it is designed for single node without requiring communicating information. The library is started by

```
ierr=talsh_init(buf_size)
```

There are two modes to initialize the library. Either the buffer size (buf_size) is provided by the user and the library only uses the given amount of memory, or the arrays are allocated dynamical and use the memory they require.

The tensors are created by the command

```
ierr=talsh_tensor_construct(tensor,C8,dims,init_val=ZERO)
```

, where tensor is a object of the type talsh_tens_t, C8 defines the datatype, dims defines the size of the tensor and is a one dimensional array of integers. These tensors can be initialized during creation with the optional parameter init_val.

By using pointers the tensors can be accessed by following commands

```
ierr=talsh_tensor_get_body_access(tensor,body_p,C8,0,DEV_HOST)
call c_f_pointer(body_p,tens,dims)
```

. The pointer is set to the position of the body of the tensor with the first command. The C pointer (C_PTR) body_p is then used to link the fortran array tens to a tensor of the dimensions dims. This enables the initialization of tensors, operations on the elements and extraction of elements. It is possible to use external libraries, e. g. InteRest in these executions.

The contractions are defined by a string for the input tensors t2 and vvvv, and the output tensor s2

```
ierr=talsh_tensor_contract("S(a,b,i,j)+=V(a,b,c,d)*T(c,d,i,j)",s2,vvvv,t2,scale=NUM)
```

. The result can be scaled by a number NUM.

```
ierr=talsh_tensor_destruct(tensor)
```

is used to delete the tensors and release the memory.

9 Integral transformations

The integral transformation takes some time, especially in the MOLTRA module in DIRAC. An efficient implementation is desired and for this reason several approaches were tested and compared. In the ExaTENSOR part of ExaCorr four different integral transformation schemes are available. The 1st, 3rd, and 4th follow equations 18-21 in the main text. The latter two reuse the half transformed integrals which increases efficiency. The 4th scheme uses batches to avoid the large AO tensor. An alternative is to construct the density matrix and use it for the transformation (scheme 2):

$$D_{\kappa p \lambda q} = C^{\dagger}_{\kappa p} C_{\lambda q}, \tag{41}$$

$$[pq|\mu\nu] = \sum_{\lambda\kappa}^{n_{AO}} D_{\kappa p \lambda q} \left[\kappa\lambda|\mu\nu\right], \tag{42}$$

$$[pq|rs] = \sum_{\mu\nu}^{n_{AO}} D_{\mu r \nu s} \left[pq|\mu\nu\right] \tag{43}$$

This scheme has a N^6 scaling and the density matrix requires additional memory as well. It is meant as a precursor for algorithms that exploit sparsity in the density matrices. The 3rd and 4th scheme are currently the best performing ones and scheme 4 is taken as a default as it has the smallest memory footprint.

Table 11: Compare the time in seconds (t_I) of different integral transformation schemes for different systems alongside the time for the CCSD computations (t_{CC}). The number of occupied (n_{occ}) and virtual (n_{vir}) spinors are listed in the table. n(nodes) is the number of used summit nodes.

system	n_{occ}	n_{vir}	n(nodes)	t_I (1)	t_I (2)	t_I (3)	t_I (4)	t_{CC}
SF6 (DZ)	48	252	8	56	56	39	62	$\approx$ 380
SF6 (TZ)	48	458	32	232	356	152	209	$\approx$ 1050
LaF3 (DZ)	50	240	8	129	129	60	129	$\approx$ 300
UF6 (DZ)	66	314	40	430	477	219	277	$\approx$ 2000

References

(1) DIRAC, a relativistic ab initio electronic structure program, Release DIRAC19 (2019), written by A. S. P. Gomes, T. Saue, L. Visscher, H. J. Aa. Jensen, and R. Bast, with contributions from I. A. Aucar, V. Bakken, K. G. Dyall, S. Dubillard, U. Ekström, E. Eliav, T. Enevoldsen, E. Faßhauer, T. Fleig, O. Fossgaard, L. Halbert, E. D. Hedegård, B. Heimlich–Paris, T. Helgaker, J. Henriksson, M. Iliaš, Ch. R. Jacob, S. Knecht, S. Komorovský, O. Kullie, J. K. Lærdahl, C. V. Larsen, Y. S. Lee, H. S. Nataraj, M. K. Nayak, P. Norman, G. Olejniczak, J. Olsen, J. M. H. Olsen, Y. C. Park, J. K. Pedersen, M. Pernpointner, R. di Remigio, K. Ruud, P. Sałek, B. Schimmelpfennig, B. Senjean, A. Shee, J. Sikkema, A. J. Thorvaldsen, J. Thyssen, J. van Stralen, M. L. Vidal, S. Villaume, O. Visser, T. Winther, and S. Yamamoto (available at http://dx.doi.org/10.5281/zenodo.3572669, see also http://www.diracprogram.org).

(2) Visscher, L.; Lee, T. J.; Dyall, K. G. Formulation and implementation of a relativistic unrestricted coupled-cluster method including noniterative connected triples. *J. Chem. Phys.* **1996**, *105*, 8769–8776.

(3) Christiansen, O.; Koch, H.; Jørgensen, P. The second-order approximate coupled cluster singles and doubles model CC2. *Chemical Physics Letters* **1995**, *243*, 409–418.

(4) Deegan, M. J. O.; Knowles, P. J. Perturbative corrections to account for triple excita-

tions in closed and open shell coupled cluster theories. *Chem. Phys. Lett.* **1994**, *227*, 321–326.

(5) Shee, A.; Visscher, L.; Saue, T. Analytic one-electron properties at the 4-component relativistic coupled cluster level with inclusion of spin-orbit coupling. *J. Chem. Phys.* **2016**, *145*, 184107.

(6) Jamshidi, Z.; Lushchikova, O. V.; Bakker, J. M.; Visscher, L. Not Completely Innocent: How Argon Binding Perturbs Cationic Copper Clusters. *J. Phys. Chem. A* **2020**, *124*, 9004–9010.

(7) Zmbov, K. F.; Margrave, J. L. Mass Spectrometric Studies of Scandium Yttrium Lanthanum and Rare-earth Fluorides. *Adv. Chem. Ser.* **1968**, 267.

(8) Zmbov, K. F.; Margrave, J. L. Mass Spectrometric Studies at High Temperatures .13. Stabilities of Samarium Europium and Gadolinium Mono- and Difluorides. *J. Inorg. Nucl. Chem.* **1967**, *29*, 59.

(9) Kimura, M.; Schomaker, V.; Smith, D. W.; Weinstock, B. Electron-Diffraction Investigation of the Hexafluorides of Tungsten, Osmium, Iridium, Uranium, Neptunium, and Plutonium. *J. Chem. Phys.* **1968**, *48*, 4001–4012.

(10) Repisky, M. InteRest, An integral library for relativistic quantum chemistry. 2018.
